# Chinese and global burdens of gastric cancer from 1990 to 2019

**DOI:** 10.1002/cam4.3892

**Published:** 2021-05-01

**Authors:** Yuxin He, Yida Wang, Fujuan Luan, Zhuwen Yu, Huang Feng, Bingxin Chen, Weichang Chen

**Affiliations:** ^1^ Department of Gastroenterology The First Affiliated Hospital of Soochow University Suzhou P. R. China; ^2^ Key Laboratory of Bio‐Resource and Eco‐Environment of Ministry of Education College of Life Sciences Sichuan University Chengdu P. R. China

**Keywords:** China, disability‐adjusted years, epidemiology, gastric cancer

## Abstract

**Background:**

Gastric cancer is a common cancer in China. This project investigated the disease burden of gastric cancer from 1990 to 2019 in China and globally.

**Methods:**

The global age‐standardized rates (ASRs) were extracted from the Global Burden of Disease. Moreover, the estimated annual percentage changes (eAPCs) in the ASRs of incidence (ASIR), mortality (ASMR), and disability‐adjusted life‐years (DALYs) were calculated to determine the trends by countries and regions.

**Results:**

In China, the ASIR declined from 37.56 to 30.64 per 100,000 and the ASMR declined from 37.73 to 21.72 per 100,000. The global ASIR decreased from 22.44 to 15.59 and the ASMR declined from 20.48 to 11.88 per 100,000 persons from 1990 to 2019. The ASIR was the lowest in Malawi (3.28 per 100,000) and the highest in Mongolia (43.7 per 100,000), whereas the ASMR was the lowest in the United States of America (3.40 per 100,000) and the highest in Mongolia (40.04 per 100,000) in 2019. The incidence of early‐onset gastric cancer increased in China. The DALYs attributed to gastric cancer presented a slight decrease during the period. China had a higher mortality/incidence ratio (0.845) and 5‐year prevalence (27.6/100,000) than most developed countries.

**Conclusion:**

China presented a steady decline in the incidence and mortality rates for gastric cancer. The global ASIR, ASMR, and DALYs showed a slight rise decrease. Different patterns of gastric cancer rates and temporal trends have been identified in different geographical regions, indicating that specific strategies are needed to prevent the increase in some countries.

## Graphical abstract

This is a report of epidemiologic characterization of gastric cancer in China and globally. This research offers an overview of differential patterns of gastric cancer rates and temporal trends, providing policy‐makers with information needed to forestall the increase in gastric cancer.

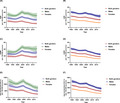

## 1 INTRODUCTION

Over the last four decades, China has experienced rapid demographic and epidemiological transitions. Rapid industrialization, urbanization, aging, and changes in lifestyle have led to a shift in the burden of the disease spectrum from infectious diseases to non‐communicable diseases.^1^ China has been an important contributor to the global cancer burden because of its large population.

Gastric cancer is a common cancer worldwide. One in 78 women and one in 33 men develop gastric cancer over their lifetime.^2^ According to GBD 2019, the DALYs in China accounted for 44.21% of the total number.^3^ Gastric cancer is the second most frequently diagnosed cancer and the second leading cause of cancer‐related deaths in China.^4^ The disease burden of gastric cancer in China is high. Comparison of the gastric cancer metrics and trends in China and other countries is of great value for developing various health policies. Moreover, reports on the spatial distribution and trends in gastric cancer would help policy makers allocate resources properly.

In this study, we extracted age‐standardized rates (ASRs) for gastric cancer from the Global Burden of Disease (GBD) study. The trends of ASRs could be a surrogate for the changing disease patterns and reflect the shift in risk factors. The estimated annual percentage changes (eAPCs) are broadly used to measure time trends in ASRs. To provide an updated picture of epidemiological trends and geographic patterns of gastric cancer, we considered temporal variations in incidence, mortality rates, disability‐adjusted life‐years (DALYs), and APCs according to age, sex, and region in China and the world.

## 2 METHODS

### 2.1 Data sources

#### 2.1.1 The Global Burden of Disease Project

The Global Burden of Disease (GBD) project presents the cancer incidence, mortality, years of life lost (YLLs), years lived with disability (YLDs), and disability‐adjusted life‐years (DALYs).^3^ The GBD project collected and analyzed the data for more than 350 diseases and injuries in 195 countries. The Global Health Data Exchange (GHDx) online data source query tool^5^ provides comparisons by age, sex, age groups, and geography from 1990 till date. This project helps visualize health trends over time as well as develop cancer control strategies to achieve global targets and improve equity in cancer care.

We collected the incidence, mortality, and DALYs of gastric cancer, and measured ASRs from 1990 to 2019 according to sex, five socio‐demographic indices (SDI), 21 regions, and 195 countries/territories. The five SDI categories (low, low‐middle, middle, high‐middle, and high SDI) are also described by the GBD project 2017.^6^ The ASRs per 100,000 person‐years are calculated using WHO world standard population.^7^

### 2.2 Statistical analyses

We gleaned data from the GBD database to examine the epidemiology trends in gastric cancer from 1990 to 2019. Only the age‐specific rates in 2019 were incorporated into the study. The ASRs from GBD were calculated using the WHO world standard population.

The estimated APCs were calculated as follows:

$$\ln\left(\mathrm{ASR}\right)=a+bx+e,\;EstimatedAPCs=100\times \left(\exp\left(b\right)‐1\right),$$

Where *x* refers to the calendar year.

The R program (R x3.5.1) was used for statistical analyses and preparing the plots.

## 3 RESULTS

### 3.1 Incidence of gastric cancer

**FIGURE 1:**
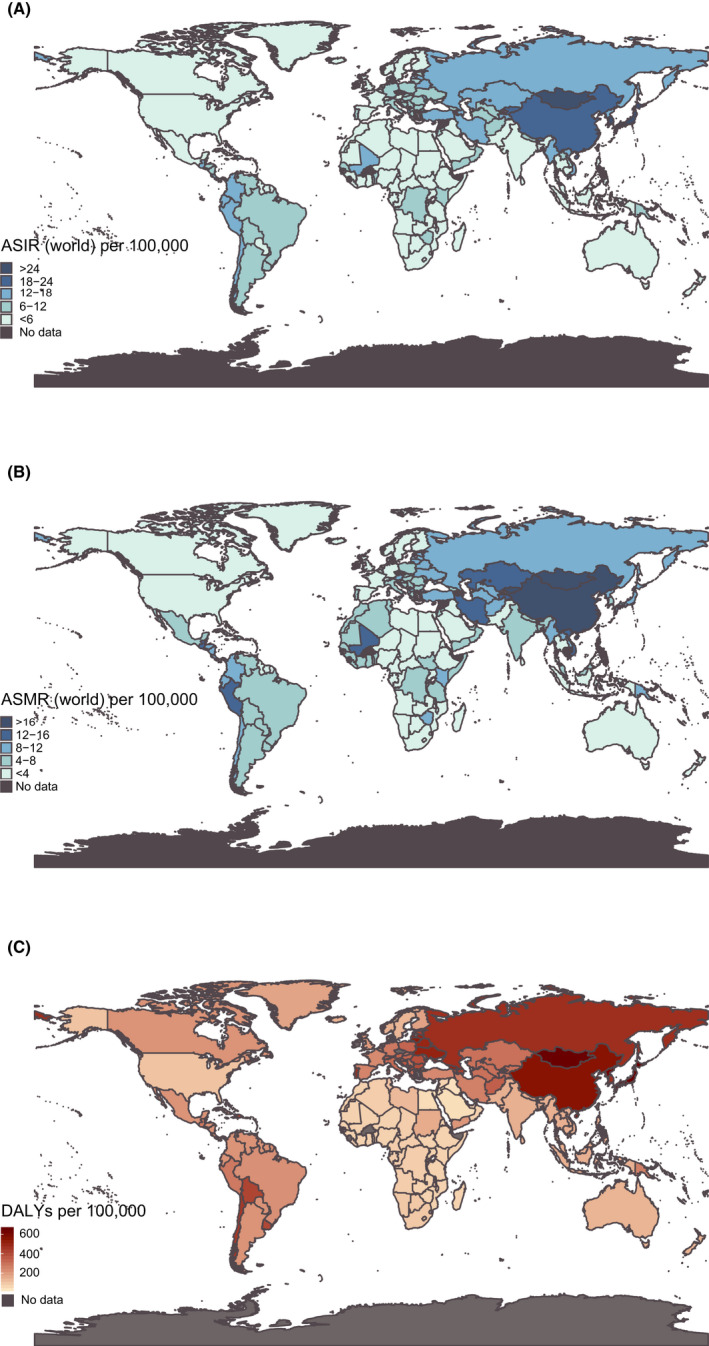
World map of ASRs for gastric cancer in different countries and territories in 2019. (A) The ASIR of gastric cancer in 2019. (B) The ASMR of gastric cancer in 2019. (C) The age‐standardized DALYs of gastric cancer in 2019

**FIGURE 2:**
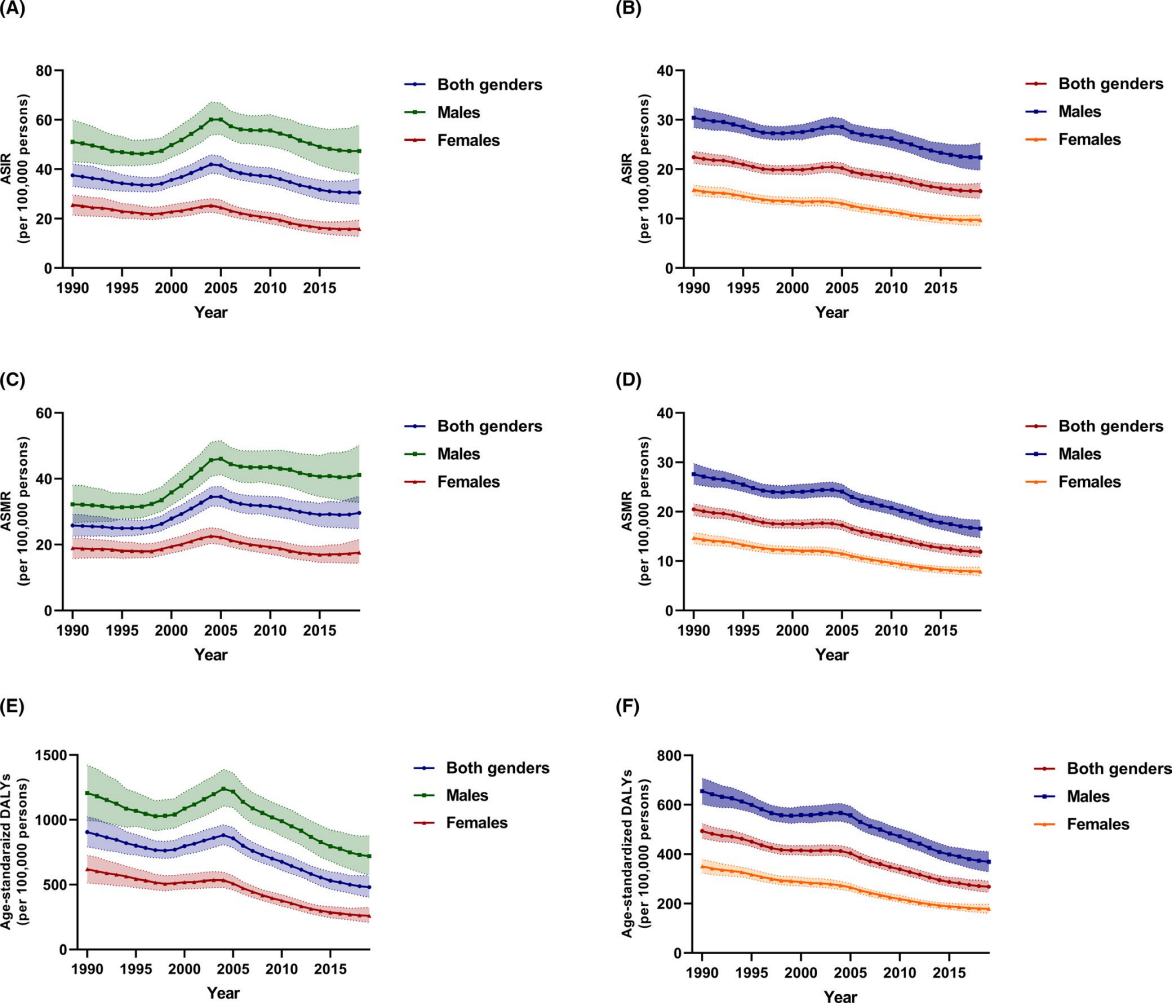
The change trends of ASRs in gastric cancer by sex in China and globally from 1990 to 2019. (A) ASIR, China. (B) ASIR, world. (C) ASMR, China. (D) ASMR, world. (E) age‐standardized DALYs, China. (F) age‐standardized, world

The incident cases in China presented a similar trend, increasing from 317,340 cases (95% UI = 277,900–359,320 cases) in 1990 to 612,820 cases (95% UI = 513,000–728,890 cases) in 2019 (Table 1). In 2019, Mainland China had a higher ASIR (30.64/100,000, 95% CI = 25.82–36.15/100,000) than the world average (15.59/100,000, 95% CI = 14.11–17.15/100,000) (Figure 1A; Table 2). ASIR decreased in Mainland China (eAPC = −0.18, 95% CI = −0.33 to 0.01) from 1990 to 2019. The ASIR for males remained stable (eAPC = −0.07, 95% CI = −0.29 to 0.23) during the study period, meanwhile the indicator experienced a downward tendency for females (eAPC = −0.38, 95% CI = −0.53 to −0.20) (Table 2; Table S1; Figure 2A).

**TABLE 1:** The incidence, death, DALY, and their change trends of stomach cancer from 1990 to 2019

Characteristics	Incident cases (NO.)					Deaths (NO.)					DALYS (NO.)				
	1990		2019		1990–2019 increase (%)	1990		2019		1990–2019 increase (%)	1990		2019		1990–2019 increase (%)
	Both (95% UI) (No.×1000)	Male/ Female ratio	Both (95% UI) (No.×1000)	Male/ Female ratio		Both (95% UI) (No.×1000)	Male/ Female ratio	Both (95% UI) (No.×1000)	Male/ Female ratio		Both (95% UI) (No.×1000)	Male/ Female ratio	Both (95% UI) (No.×1000)	Male/ Female ratio	
Global	883.40 (834.24,929.17)	1.63	1269.81 (1150.49,1399.82)	2.00	43.74%	788.32 (742.79,834.00)	1.57	957.19 (870.95,1034.65)	1.77	21.42%	20463.03 (19234.88,21737.50)	1.67	22220.98 (20301.49,24071.76)	1.89	8.59%
China	317.34 (277.90,359.32)	1.89	612.82 (513.00,728.89)	2.79	93.11%	305.47 (267.21,345.40)	1.82	421.54 (353.52,493.18)	2.43	38.00%	8248.79 (7173.64,9366.34)	1.91	9824.99 (8191.72,11632.86)	2.65	19.11%
SDI															
Low SDI	26.77 (23.56,29.64)	1.49	41.31 (37.01,45.96)	1.21	54.33%	27.14 (24.01,29.95)	1.52	43.88 (39.31,48.90)	1.32	61.64%	789.50 (692.37,877.16)	1.38	1233.25 (1093.36,1390.35)	1.27	56.21%
Low‐middle SDI	85.80 (78.53,92.35)	1.38	148.56 (135.58,162.71)	1.35	73.15%	85.48 (78.26,91.91)	1.38	141.96 (130.15,154.60)	1.28	66.07%	2502.37 (2287.77,2693.83)	1.32	3773.51 (3441.56,4132.70)	1.29	50.80%
Middle SDI	251.42 (226.67,277.98)	1.71	396.75 (349.28,452.22)	1.85	57.81%	244.35 (221.14,268.69)	1.66	345.27 (305.03,387.65)	2.00	41.30%	6689.51 (6009.34,7370.87)	1.72	8273.27 (7276.66,9296.06)	2.14	23.68%
High‐middle SDI	291.59 (275.03,308.00)	1.67	380.86 (337.20,427.02)	2.27	30.61%	276.44 (260.10,292.70)	1.60	280.96 (251.41,308.75)	2.01	1.63%	7012.14 (6590.59,7450.79)	1.80	6352.75 (5662.39,7023.40)	2.24	−9.40%
High SDI	227.56 (219.66,231.99)	1.64	237.76 (209.74,260.86)	1.78	4.48%	154.66 (148.23,157.91)	1.49	144.79 (128.82,154.03)	1.58	−6.38%	3463.38 (3371.53,3517.20)	1.67	2579.96 (2390.87,2704.47)	1.85	−25.51%
Region															
Andean Latin America	5.98 (5.44,6.54)	1.25	12.37 (10.07,15.03)	1.26	107.06%	6.20 (5.65,6.79)	1.23	11.79 (9.65,14.22)	1.13	90.10%	156.48 (142.49,171.50)	1.26	263.03 (212.83,321.88)	1.19	68.09%
Australasia	2.38 (2.28,2.48)	1.69	3.45 (2.79,4.21)	1.74	44.78%	1.76 (1.68,1.82)	1.56	2.05 (1.84,2.22)	1.57	16.16%	37.55 (36.25,38.68)	1.74	38.91 (35.98,41.63)	1.73	3.62%
Caribbean	2.92 (2.67,3.10)	1.61	4.36 (3.76,4.97)	1.52	48.95%	2.93 (2.67,3.11)	1.58	4.12 (3.56,4.72)	1.47	40.38%	71.17 (63.86,76.22)	1.57	97.70 (82.77,113.47)	1.54	37.27%
Central Asia	13.39 (12.98,13.76)	1.64	12.13 (10.99,13.40)	1.74	−9.45%	13.18 (12.77,13.54)	1.59	11.64 (10.55,12.81)	1.72	−11.70%	374.34 (362.96,384.62)	1.79	324.97 (294.10,359.73)	1.81	−13.19%
Central Europe	26.46 (25.81,26.92)	1.71	21.72 (19.11,24.41)	1.78	−17.92%	26.33 (25.63,26.81)	1.66	20.11 (17.72,22.59)	1.72	−23.61%	627.38 (613.87,637.67)	1.89	428.30 (374.97,482.89)	1.99	−31.73%
Central Latin America	15.50 (14.96,15.89)	1.22	30.51 (25.95,35.82)	1.19	96.88%	15.45 (14.84,15.86)	1.23	27.30 (23.37,31.87)	1.23	76.74%	388.39 (376.95,397.12)	1.28	647.87 (550.78,763.65)	1.28	66.81%
Central Sub‐Saharan Africa	2.72 (2.21,3.29)	1.72	4.25 (3.38,5.31)	1.60	55.97%	2.75 (2.30,3.26)	1.77	4.28 (3.42,5.31)	1.58	55.42%	81.39 (66.71,97.90)	1.67	125.50 (97.76,157.85)	1.64	54.20%
East Asia	325.71 (285.49,367.29)	1.89	626.49 (526.59,741.27)	2.77	92.34%	313.10 (274.77,353.49)	1.81	432.99 (364.16,504.15)	2.40	38.29%	8466.87 (7396.55,9577.28)	1.90	10102.78 (8469.06,11888.73)	2.63	19.32%
Eastern Europe	87.03 (84.19,88.71)	1.35	54.12 (48.79,59.79)	1.44	−37.82%	80.93 (78.37,82.54)	1.28	45.01 (40.63,49.87)	1.38	−44.39%	2123.34 (2045.47,2165.63)	1.53	1081.23 (971.54,1201.16)	1.60	−49.08%
Eastern Sub‐Saharan Africa	8.23 (7.15,9.17)	1.46	11.76 (10.23,13.42)	1.41	42.77%	8.34 (7.25,9.30)	1.51	12.08 (10.58,13.75)	1.38	44.94%	247.21 (212.39,277.39)	1.34	345.78 (298.17,399.76)	1.35	39.87%
High‐income Asia Pacific	123.73 (119.61,126.50)	1.81	128.17 (108.45,147.69)	1.91	3.59%	69.65 (66.94,71.22)	1.69	69.86 (59.75,75.61)	1.72	0.30%	1712.44 (1668.47,1744.70)	1.77	1161.35 (1046.33,1234.34)	2.04	−32.18%
High‐income North America	30.32 (29.09,31.12)	1.49	37.58 (32.97,42.69)	1.64	23.96%	21.41 (20.33,22.01)	1.37	22.32 (20.68,23.32)	1.45	4.25%	441.37 (427.97,450.28)	1.59	446.19 (426.86,461.36)	1.64	1.09%
North Africa and Middle East	23.49 (20.47,25.67)	1.72	42.26 (38.24,46.71)	1.69	79.94%	23.54 (20.40,25.76)	1.72	39.61 (35.88,43.88)	1.68	68.22%	653.67 (567.33,717.36)	1.63	1011.45 (904.54,1132.98)	1.61	54.73%
Oceania	0.42 (0.33,0.51)	2.15	0.94 (0.71,1.19)	2.07	123.40%	0.41 (0.32,0.50)	2.10	0.91 (0.69,1.14)	2.00	121.60%	12.86 (9.95,15.83)	2.16	28.67 (21.58,36.73)	2.12	123.02%
South Asia	57.57 (51.98,63.22)	1.24	99.40 (87.31,113.63)	1.05	72.65%	57.25 (51.44,63.01)	1.26	99.07 (86.31,112.44)	1.04	73.06%	1745.77 (1561.47,1915.36)	1.16	2774.51 (2413.91,3168.20)	1.02	58.93%
Southeast Asia	28.07 (24.31,31.09)	1.40	40.06 (35.46,44.83)	1.57	42.70%	28.21 (24.57,31.06)	1.39	38.21 (34.17,42.46)	1.50	35.46%	799.97 (696.91,879.26)	1.36	987.32 (874.36,1105.38)	1.61	23.42%
Southern Latin America	8.46 (8.19,8.70)	1.91	10.69 (8.56,13.33)	1.85	26.47%	8.52 (8.22,8.77)	1.82	9.95 (9.29,10.52)	1.72	16.82%	195.88 (190.34,201.20)	2.06	209.27 (198.03,220.24)	1.97	6.83%
Southern Sub‐Saharan Africa	2.42 (2.20,2.63)	1.39	3.61 (3.28,3.97)	1.30	49.25%	2.45 (2.23,2.66)	1.35	3.66 (3.35,4.01)	1.25	49.11%	67.92 (62.30,73.46)	1.48	95.72 (86.52,106.65)	1.43	40.92%
Tropical Latin America	16.11 (15.50,16.60)	1.98	24.54 (23.07,25.65)	1.82	52.31%	16.20 (15.49,16.72)	1.93	23.45 (21.82,24.58)	1.76	44.73%	421.05 (405.35,433.82)	2.05	556.71 (530.26,581.24)	1.87	32.22%
Western Europe	93.95 (90.02,96.14)	1.38	86.45 (75.20,97.34)	1.49	−7.98%	80.78 (77.14,82.72)	1.28	63.12 (57.06,66.79)	1.34	−21.86%	1607.13 (1557.22,1636.77)	1.52	1097.49 (1030.85,1149.17)	1.60	−31.71%
Western Sub‐Saharan Africa	8.53 (7.40,9.62)	1.49	14.96 (12.91,17.37)	1.36	75.45%	8.92 (7.79,10.04)	1.50	15.66 (13.59,17.95)	1.36	75.66%	230.85 (199.59,263.34)	1.47	396.23 (337.14,463.23)	1.36	71.64%

Abbreviations: DALY, disability‐adjusted life year; SDI, socio‐demographic index; UI, uncertain interval.

**TABLE 2:** The ASRs and variations of stomach cancer from 1990 to 2017

Characteristics	ASIR (per 100,000 persons)					ASMR (per 100,000 persons)					Age‐Standardized DALY Rate (per 100,000 persons)				
	1990 (95% CI)		2019 (95% CI)		eAPC (95% CI)	1990 (95% CI)		2019 (95% CI)		eAPC (95% CI)	1990 (95% CI)		2019 (95% CI)		eAPC (95% CI)
	Both	Male/ Female ratio	Both	Male/ Female ratio		Both	Male/ Female ratio	Both	Male/ Female ratio		Both	Male/ Female ratio	Both	Male/ Female ratio	
Global	22.44 (21.21,23.59)	1.92	15.59 (14.11,17.15)	2.31	−0.31 (−0.37,−0.23)	20.48 (19.25,21.62)	1.88	11.88 (10.82,12.82)	2.09	−0.42 (−0.47,−0.36)	493.38 (463.73,523.70)	1.87	268.40 (245.49,290.61)	2.07	−0.46 (−0.51,−0.40)
China	37.56 (33.08,42.27)	2.00	30.64 (25.82,36.15)	3.00	−0.18 (−0.33,0.01)	37.73 (33.20,42.39)	1.96	21.72 (18.31,25.31)	2.72	−0.42 (−0.53,−0.30)	905.54 (791.75,1024.49)	1.95	481.15 (403.20,567.36)	2.75	−0.47 (−0.57,−0.35)
SDI															
Low SDI	11.33 (10.01,12.51)	1.53	8.01 (7.23,8.84)	1.31	−0.29 (−0.35,−0.22)	12.09 (10.78,13.28)	1.56	8.94 (8.09,9.89)	1.43	−0.26 (−0.32,−0.19)	296.61 (261.86,327.96)	1.44	211.99 (189.09,237.06)	1.37	−0.29 (−0.35,−0.21)
Low‐middle SDI	14.16 (12.98,15.20)	1.38	10.92 (9.97,11.94)	1.46	−0.23 (−0.30,−0.14)	14.81 (13.54,15.91)	1.38	10.79 (9.86,11.75)	1.41	−0.27 (−0.34,−0.19)	371.10 (340.05,399.43)	1.33	259.64 (237.33,283.81)	1.38	−0.30 (−0.37,−0.22)
Middle SDI	24.50 (22.18,27.08)	1.80	16.22 (14.33,18.42)	2.01	−0.34 (−0.43,−0.23)	24.85 (22.60,27.23)	1.76	14.63 (12.98,16.34)	2.21	−0.41 (−0.49,−0.32)	597.68 (539.40,656.25)	1.78	323.39 (285.17,362.95)	2.28	−0.46 (−0.53,−0.37)
High‐middle SDI	27.30 (25.74,28.82)	2.16	18.76 (16.62,21.02)	2.76	−0.31 (−0.39,−0.22)	26.38 (24.76,27.91)	2.13	13.85 (12.41,15.21)	2.55	−0.48 (−0.53,−0.41)	636.28 (597.50,675.77)	2.19	314.06 (280.11,346.89)	2.57	−0.51 (−0.57,−0.44)
High SDI	21.99 (21.24,22.41)	2.22	12.40 (11.06,13.58)	2.25	−0.44 (−0.48,−0.38)	14.83 (14.21,15.14)	2.14	7.18 (6.50,7.59)	2.16	−0.52 (−0.54,−0.50)	343.24 (334.59,348.46)	2.08	145.30 (136.59,151.74)	2.12	−0.58 (−0.59,−0.56)
Regions															
Andean Latin America	29.65 (26.92,32.44)	1.33	22.43 (18.30,27.24)	1.40	−0.24 (−0.38,−0.07)	31.65 (28.85,34.63)	1.29	21.51 (17.55,25.91)	1.25	−0.32 (−0.44,−0.17)	716.00 (650.82,783.72)	1.34	461.18 (373.90,562.48)	1.28	−0.36 (−0.48,−0.20)
Australasia	10.17 (9.70,10.57)	2.16	7.00 (5.66,8.53)	2.03	−0.31 (−0.44,−0.16)	7.58 (7.20,7.86)	2.08	4.01 (3.64,4.32)	1.91	−0.47 (−0.50,−0.44)	161.78 (156.20,166.61)	2.05	84.50 (78.68,89.98)	1.90	−0.48 (−0.51,−0.44)
Caribbean	11.30 (10.32,11.97)	1.75	8.44 (7.28,9.63)	1.73	−0.25 (−0.35,−0.15)	11.54 (10.53,12.21)	1.73	7.97 (6.90,9.13)	1.69	−0.31 (−0.40,−0.21)	265.63 (239.02,284.40)	1.71	189.55 (160.41,220.08)	1.69	−0.29 (−0.39,−0.17)
Central Asia	28.00 (27.11,28.78)	2.28	16.37 (14.95,17.94)	2.24	−0.42 (−0.47,−0.36)	28.11 (27.18,28.91)	2.26	16.33 (14.91,17.86)	2.26	−0.42 (−0.47,−0.36)	748.11 (724.79,769.16)	2.33	400.85 (364.02,442.66)	2.22	−0.46 (−0.52,−0.41)
Central Europe	18.06 (17.57,18.39)	2.27	10.29 (9.03,11.58)	2.39	−0.43 (−0.49,−0.36)	18.18 (17.62,18.53)	2.25	9.38 (8.25,10.54)	2.41	−0.48 (−0.54,−0.43)	424.85 (415.55,431.68)	2.36	215.06 (187.81,242.35)	2.45	−0.49 (−0.56,−0.43)
Central Latin America	19.01 (18.16,19.56)	1.31	13.01 (11.08,15.25)	1.40	−0.32 (−0.41,−0.20)	19.69 (18.70,20.31)	1.31	11.79 (10.11,13.75)	1.46	−0.40 (−0.49,−0.30)	434.34 (420.65,444.79)	1.37	268.37 (228.66,315.56)	1.48	−0.38 (−0.47,−0.27)
Central Sub‐Saharan Africa	11.90 (9.86,14.11)	1.97	7.95 (6.49,9.82)	1.93	−0.33 (−0.48,−0.16)	12.72 (10.78,14.86)	2.00	8.47 (6.95,10.43)	1.94	−0.33 (−0.47,−0.18)	313.58 (261.08,371.43)	1.96	204.11 (163.10,253.38)	1.93	−0.35 (−0.49,−0.18)
East Asia	37.11 (32.73,41.70)	2.00	30.24 (25.55,35.54)	2.98	−0.19 (−0.33,0.00)	37.23 (32.83,41.74)	1.96	21.51 (18.23,24.95)	2.70	−0.42 (−0.52,−0.30)	895.01 (784.78,1009.82)	1.95	477.93 (402.48,560.38)	2.74	−0.47 (−0.56,−0.35)
Eastern Europe	30.90 (29.83,31.50)	2.33	16.07 (14.47,17.76)	2.25	−0.48 (−0.53,−0.42)	28.87 (27.93,29.48)	2.33	13.17 (11.90,14.60)	2.31	−0.54 (−0.59,−0.50)	754.96 (725.79,770.39)	2.39	330.40 (297.06,366.60)	2.30	−0.56 (−0.61,−0.52)
Eastern Sub‐Saharan Africa	10.68 (9.34,11.84)	1.61	7.19 (6.35,8.15)	1.59	−0.33 (−0.40,−0.23)	11.33 (9.90,12.56)	1.65	7.78 (6.88,8.78)	1.57	−0.31 (−0.39,−0.22)	286.46 (249.24,320.37)	1.51	184.39 (160.88,210.64)	1.54	−0.36 (−0.43,−0.26)
High‐income Asia Pacific	61.54 (59.31,62.98)	2.33	28.20 (24.20,32.29)	2.47	−0.54 (−0.60,−0.48)	35.52 (33.98,36.40)	2.26	13.99 (12.38,14.96)	2.51	−0.61 (−0.64,−0.58)	839.67 (816.85,855.98)	2.11	284.70 (262.80,300.26)	2.34	−0.66 (−0.68,−0.64)
High‐income North America	8.52 (8.19,8.73)	2.13	6.12 (5.38,6.95)	1.99	−0.28 (−0.37,−0.18)	5.96 (5.68,6.12)	2.04	3.51 (3.29,3.66)	1.85	−0.41 (−0.43,−0.39)	129.30 (125.59,131.82)	2.08	77.38 (74.42,79.93)	1.84	−0.40 (−0.42,−0.38)
North Africa and Middle East	13.75 (11.92,15.01)	1.72	10.10 (9.14,11.14)	1.66	−0.26 (−0.34,−0.15)	14.42 (12.52,15.77)	1.72	9.89 (8.91,10.89)	1.65	−0.31 (−0.39,−0.21)	345.46 (298.29,379.46)	1.65	218.12 (196.87,242.38)	1.60	−0.37 (−0.44,−0.27)
Oceania	13.77 (11.02,16.60)	1.97	12.88 (10.10,15.85)	1.84	−0.07 (−0.22,0.14)	14.49 (11.65,17.37)	1.92	13.40 (10.57,16.47)	1.78	−0.07 (−0.22,0.12)	360.34 (281.99,441.07)	2.01	335.01 (255.86,422.08)	1.93	−0.07 (−0.24,0.15)
South Asia	9.94 (8.92,10.96)	1.19	7.01 (6.16,7.97)	1.10	−0.29 (−0.39,−0.18)	10.47 (9.35,11.54)	1.22	7.24 (6.32,8.23)	1.10	−0.31 (−0.40,−0.20)	264.88 (237.52,291.51)	1.12	182.05 (158.68,207.51)	1.05	−0.31 (−0.41,−0.20)
Southeast Asia	10.91 (9.46,12.05)	1.62	6.72 (5.97,7.50)	1.83	−0.38 (−0.46,−0.29)	11.46 (10.00,12.62)	1.62	6.68 (6.00,7.39)	1.79	−0.42 (−0.48,−0.33)	280.51 (244.73,308.55)	1.57	153.52 (136.49,171.65)	1.80	−0.45 (−0.52,−0.37)
Southern Latin America	18.54 (17.93,19.07)	2.41	12.83 (10.24,16.01)	2.42	−0.31 (−0.45,−0.14)	18.96 (18.24,19.55)	2.33	11.83 (11.07,12.51)	2.35	−0.38 (−0.41,−0.34)	421.14 (409.33,432.43)	2.48	256.51 (242.93,269.83)	2.41	−0.39 (−0.42,−0.36)
Southern Sub‐Saharan Africa	8.74 (7.89,9.51)	1.79	6.52 (5.97,7.13)	1.78	−0.25 (−0.33,−0.16)	9.22 (8.33,10.06)	1.75	6.86 (6.32,7.47)	1.75	−0.26 (−0.33,−0.17)	223.03 (204.28,241.65)	1.85	158.41 (144.27,175.05)	1.84	−0.29 (−0.37,−0.20)
Tropical Latin America	18.06 (17.23,18.65)	2.21	10.20 (9.57,10.67)	2.23	−0.44 (−0.46,−0.41)	18.88 (17.86,19.55)	2.15	9.85 (9.11,10.33)	2.20	−0.48 (−0.50,−0.45)	432.40 (414.90,446.15)	2.28	225.67 (214.30,235.87)	2.20	−0.48 (−0.50,−0.45)
Western Europe	16.14 (15.50,16.50)	2.06	9.45 (8.23,10.67)	1.95	−0.41 (−0.49,−0.34)	13.74 (13.11,14.07)	1.99	6.48 (5.97,6.81)	1.89	−0.53 (−0.55,−0.51)	289.10 (280.93,294.19)	2.05	131.98 (125.26,137.70)	1.93	−0.54 (−0.56,−0.53)
Western Sub‐Saharan Africa	10.19 (8.89,11.43)	1.54	8.65 (7.53,9.84)	1.48	−0.15 (−0.26,−0.03)	11.13 (9.74,12.47)	1.57	9.54 (8.33,10.78)	1.48	−0.14 (−0.25,−0.03)	248.41 (217.24,281.75)	1.47	199.71 (172.02,229.87)	1.51	−0.20 (−0.31,−0.07)

Abbreviations: ASIR, age‐standardized incidence rate; ASMR, age‐standardized mortality rate; ASR, age‐standardized rate; CI, confidence interval; DALY, disability‐adjusted life year; eAPC, estimated annual percentage change; SDI, sociodemographic index.

The global incident cases of gastric cancer went upward from 883,400 cases (95% UI = 834,240 to 929,170 cases) in 1990 to 1,269,810 cases (95% UI = 1,150,490 to 1,399,820 cases) in 2019, corresponding to an increase of 43.74% (Table 1). The incident cases in all SDI quintiles went upward. The low‐middle SDI quintile had the highest increase of 73.15%, whereas the high‐SDI quintile had the lowest increase of 4.48% (Table 1).

According to GBD 2019,^3^ in 204 countries and territories, Mongolia displayed the highest ASIR of 43.7/100,000 (95% CI = 34.29–55.1/100,000), and was followed by Bolivia (34.02/100,000, 95% CI = 26.85–42.02/100,000), China (30.64/100,000, 95% CI = 25.82–36.15/100,000), South Korea (28.67/100,000, 95% CI = 23.65–34.17/100,000), and Japan (28.29/100,000, 95% CI = 23.71–33.27/100,000). The lowest ASIR was observed in Malawi (3.28/100,000, 95% CI = 2.67–3.91/100,000) (Figure 1A; Table S2). Trinidad and Tobago displayed the largest decrease (eAPC = −0.60, 95% CI = −0.70 to −0.48) from 1990 to 2019 (Table S2).

In terms of geographic regions, the ASIR was the highest in East Asia (30.24/100,000, 95% CI = 25.55–35.54/100,000) and the lowest in High‐income North America (6.12/100,000, 95% CI = 5.38–6.95/100,000) in 2019. The ASIR for males was 1.10–3.0 times higher than that for females (Table 2). Overall, the global ASIR showed a decreasing trend from 1990 to 2019 (Figure 2B), with the largest decline in High‐income Asia Pacific (eAPC =−0.54, 95% CI = −0.60 to −0.48). The ASIR performed a downward tendency in all SDI quintiles, whereas the High SDI countries exhibited the most significant decline (eAPC = −0.44, 95% CI = −0.48 to −0.38) (Table 2).

### 3.2 Mortality of gastric cancer

Gastric cancer caused 421,540 deaths (95% UI = 353,520 to 493,180) in China in 2019, corresponding to a 38% increase from the 305,470 deaths (95% UI = 267,210 to 345,400 deaths) in 1990 (Table 1). The ASMR in China showed an overall downward trend during the period (eAPC = −0.42, 95% CI = −0.47 to −0.36) (Figure 2C). The ASMR among females conducted a more conspicuous declining tendency (eAPC = −0.53, 95% CI = −0.63 to −0.40) than that of males (eAPC = −0.35, 95% CI = −0.50 to −0.16) (Table 2; Table S1).

From 1990 to 2019, the global gastric cancer deaths increased by 21.42%, from 788,320 deaths (95% UI = 742,79 to 834,000) to 957,190 deaths (95% UI = 870,950 to 1,034,650). Geographically, Oceania presented the largest increase in deaths (121.60%) while Eastern Europe took the least (−44.39%) (Table 1). Besides, the death cases showed an increasing trend among all SDI quintiles. The middle‐SDI quintile presented the highest deaths of gastric cancer (345,270 deaths, 95% UI = 305,030 to 387,650 deaths), whereas the low‐SDI quintile had the lowest (438,800 deaths, 95% UI = 393,100 to 489,000 deaths) in 2019 (Table 1).

Figure 1B shows the ASMR in different countries and territories. The ASMR ranked highest in Mongolia (40.04/100,000, 95% CI = 36.3–57.48/100,000), followed by Bolivia (36.11/100,000, 95% CI = 28.77–44.26/100,000), Afghanistan (29.3/100,000, 95% CI = 21.25–36.25/100,000), and Guatemala (27.97/100,000, 95% CI = 22.45–34.43/100,000), whereas the ASMR was lowest in the United States of America (3.40/100,000, 95% CI = 3.19–3.54/100,000). In addition, the highest decrease was demonstrated in Republic of Korea (eAPC=−0.73, 95% CI = −0.76 to‐0.70) (Table S2).

In terms of geographic regions, the ASMR was highest in Andean Latin America (21.51/100,000, 95% CI = 17.55–25.91/100,000) and East Asia (21.51/100,000, 95% CI = 18.23–24.95/100,000). Oppositely, the lowest ASMR was displayed in High‐income North America (3.51/100,000, 95% CI = 3.29–3.66/100,000) in 2019 (Table 2). Overall, the ASMR decreased in all regions from 1990 to 2019, with the most remarkable drop in High‐income Asia Pacific (eAPC=−0.61, 95% CI=−0.64 to −0.58). The ASRM declined among all SDI quintiles from 1990 to 2019. The high‐SDI countries displayed the largest decrease (APC = −0.52, 95% CI = −0.54 to −0.50). While low‐SDI quintile presented the lowest decrease (APC = −0.26, 95% CI = −0.32 to −0.19) (Table 2).

### 3.3 Disease burden of gastric cancer

Gastric cancer led to a heavy health burden in China. Gastric cancer was attributable to 22,220,980 years in 2019 worldwide, with 9,824,990 years in China. From 1990 to 2019, the DALYs of gastric cancer in China increased by 19.11%, from 8,248,790 years (95% UI = 7,173,640–9,366,340 years) in 1990 to 9,824,990 years (95% UI = 8,191,720–11,632,860 years) in 2019 (Table 1). The age‐standardized DALYs declined from 905.54/100,000 (95% CI = 791.75–1,024.49/100,000) to 481.15/100,000 (95% CI = 403.20–567.36/100,000) (eAPC = −0.47, 95% CI = −0.57 to 0.35) during the study period (Table 2; Figure 2E), which showed a more significant decrease in females (eAPC = −0.58, 95% CI = −0.67 to −0.44) (Table S1).

Kuwait showed the lowest age‐standardized DALYs (64.88/100,000 95% CI = 54.13–77.72/100,000), whereas Mongolia presented the highest (1,059.24/100,000, 95% CI = 816.79–1,351.35/100,000) in 2019. The Republic of Korea had the most significant decrease in age‐standardized DALY (eAPC = −0.78,95% CI = −0.80 to −0.75) (Figure 1C; Table S2).

In terms of geographic regions, the highest age‐standardized DALYs were reported in East Asia (477.93/100,000, 95% CI = 402.48–560.38/100,000) and the lowest in High‐income North America (77.38/100,000, 95% CI = 74.42–79.93/100,000) in 2019 (Figure 1C; Table S2). High‐income Asia Pacific experienced the greatest decline in age‐standardized DALY (eAPC = −0.66, 95% CI = −0.68 to −0.64) from 1990 to 2019 (Table 2). The low‐SDI countries showed the lowest DALY (1,233,250, 95% U I= 1,093,360–1,390,350) in 2019. Age‐standardized DALYs declined among all five SDI quintiles, with the most significant decrease showed in High‐SDI quintile (eAPC = −0.58, 95% CI = −0.59 to −0.56) during the study period (Table 2).

### 3.4 Age‐specific rates of gastric cancer in China and the world in 2019

**FIGURE 3:**
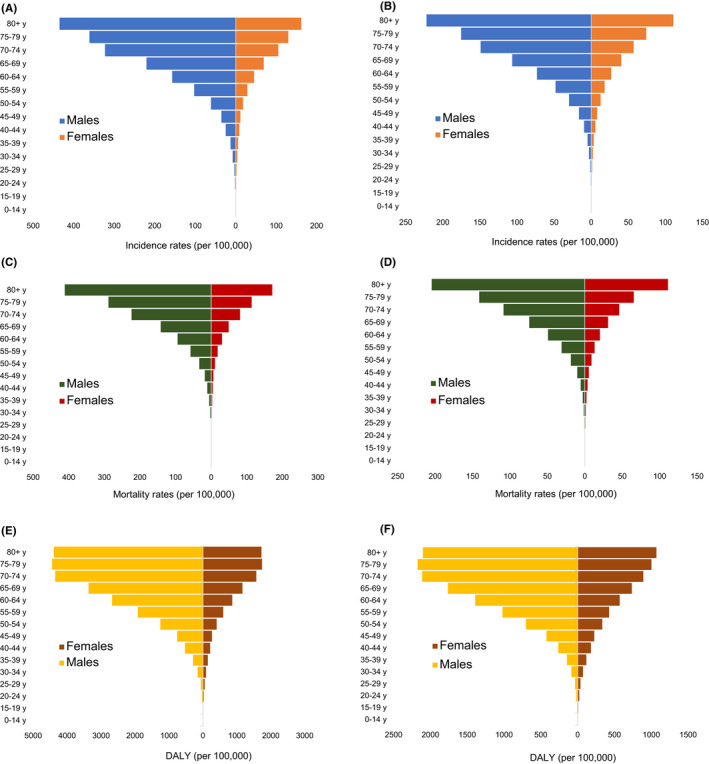
Age‐specific rates of gastric cancer by sex in China and the world in 2019. (A) Incident rate, China. (B) Incident rate, world. (C) Mortality rate, China. (D) Mortality rate, world. (E) DALYs rate, China. (F) DALYs rate, world

Figure 3 illustrates the age‐specific incidence, mortality rates, and DALY of gastric cancer in China and the world in 2019.

Overall, the age‐specific rates in China were well above the global level for each age group. In China, the incidence and mortality rates rose in parallel with age in 2019. The incidence in younger age groups (0–29 years) was less than 5/100,000, increasing sharply after 55 years. Considering the high fatality rate of gastric cancer in China, similar tendencies were observed in the age‐specific mortality rates. For patients younger than 40 years, the mortality rates were less than 5/100,000, which increased sharply after 60 years. The age‐specific DALY rate reached a high point of 1,529.10/100,000 in the 75–79 year age group (Table S3). The incidence of gastric cancer was significantly higher in males than in females among all age groups. In the subgroup analysis of gender, the age‐specific incidence and mortality rates increased with age. The age‐specific incident rates reached the peak in the 80+ year age group for both genders (435.05/100,000 for males, 161.65/100,000 for females). The mortality rates were similar to the incident rates, being higher in males. The age‐standardized DALY for males peaked in the 75–79 year age group (2173.53/100,000, 95% CI=1,951.28– 2,394.77/100,000), while it reached the peak at 80+ year age group in females (1,069.07/100,000, 95% CI=874.32–1,188.01/100,000) (Table S3; Figure 3A, 3C and 3E).

The global age‐specific rates were similar to those in China. Younger age groups (0–30 years) showed extremely low incidence (< 2/100,000) and mortality rates (< 1/100,000), with a sudden growth after 55 years. At the 80+ year age group, incidence and mortality rates reached the peak (154.03/100,000 and 147.35/100,000, respectively). The age‐specific DALY rate was highest in the 80+ year age group (1,470.51/100,000, 95% CI = 1,278.34–1,589.33/100,000) (Table S4). The age‐specific incidence rate leaped to the top of 222.10/100,000 (95% CI = 193.49–243.35/100,000) for males aged 80+ years, whereas it was 110.77/100,000 (89.33–125.23/100,000) for females aged 80+ years. The age‐specific mortality rate rocketed to the highest level in the 80+ year age group among both genders (204.46/100,000 for males and 111.06/100,000 for females). The age‐standardized DALY was highest in males aged 75–79 years (2,173.53 /100,000, 95% CI = 1,951.28–2,394.77/100,000), whereas it for females peaked in the 80+ year age group (1,069.07/100,000, 95% CI = 874.32–1,188.01/100,000) (Table S4; Figure 3B, 3D, and 3F).

### 3.5 Age‐specific trends in the incidence of gastric cancer in China

**FIGURE 4:**
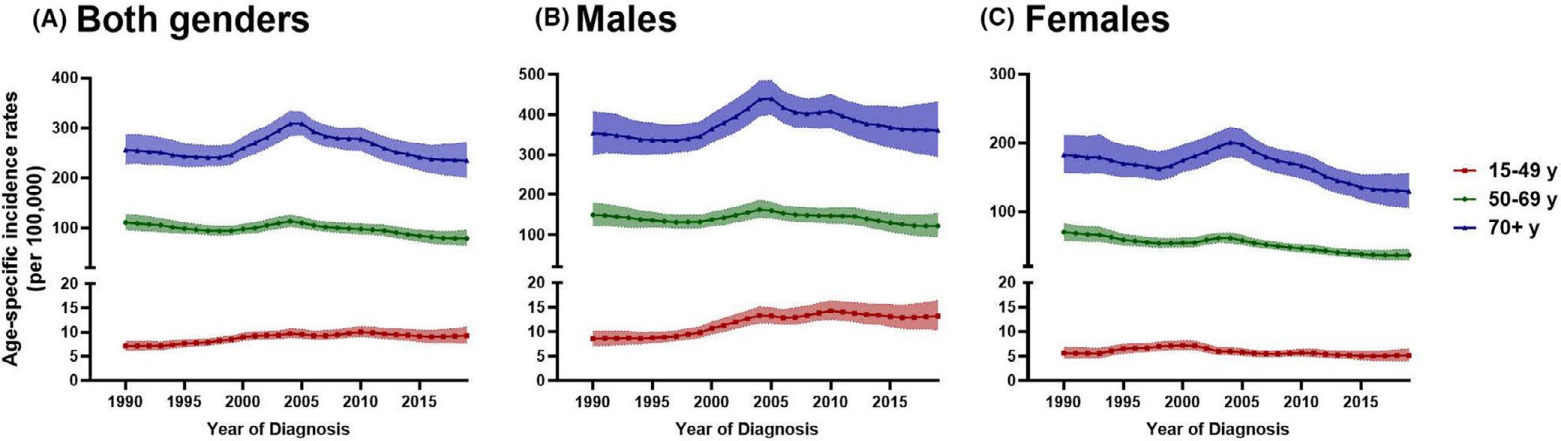
Age‐specific trends in incidence rates of gastric cancer by age group from 1990 to 2019

General trends of gastric cancer in China are shown in Figure 1A. However, there was a significant difference between genders and age groups. There was a worrisome rise in the incidence of early‐onset gastric cancer in China from 1990 to 2019. The 15–49 years age group presented an increasing trend during the study period (eAPC = 0.30, 95% CI = 0.22–0.63), whereas the 50–69 years and 70+ years age group presented a slight decrease trend from 1990 to 2019 (Figure 4; Table S5). In the subgroup analysis of gender, young men (15–49 years) were the only group to have increased incidence (eAPC = 0.54, 95% CI = 0.14–1.10). (Table S5).

## 4 DISCUSSION

This study presented a comprehensive review of the gastric cancer burden over the last 29 years based on the latest national estimated data worldwide.

This study shows a gradual decline in ASIR, ASMR, and age‐standardized DALYs in China and the world from 1990 to 2019, as well as demonstrating the variations by age and gender in China. What stands out is the V‐shaped changing trends in ASRs for the period of 1990–2005, which are quite different from the global trends. We suggest that this phenomenon is associated with the development of cancer registration in China. The National Central Cancer Registry (NCCR) of China was founded in 2002, acting as the national bureau for the management of cancer registration.^8^ Data quality of cancer registration was not high during the period of 1990–2002. There is a potential bias in the ASRs in early period.

The ASIR of gastric cancer indicated a gradual decline during the study period. In more than half of the countries examined, gastric cancer is likely to reach rare cancer thresholds by 2035.^9^ The new cases of gastric cancer kept rising year‐on‐year, but the age‐standardized incidence rate showed a downward trend in China and the world. The aging and growth of the population can explain the above phenomena.

Our results highlight a worrisome feature of a consistent increase in the incidence of early‐onset gastric cancer (EOGC) among the young population in China. The result has already been observed in both high‐incidence and low‐incidence countries.^9^, ^10^ Most early‐onset patients presented a worse prognosis than late‐onset gastric cancer patients.^10^, ^11^ Since younger individuals have less exposure to environmental carcinogens than older ones, genetic factors may play a more relevant role in EOGC than in traditional gastric cancer. This public health issue requires monitoring in the future. Further studies are needed to investigate the cause of the increasing incidence of EOGC.

Most studies reported 1.8–2.0 times higher risk of gastric cancer in men than in women.^12^, ^13^ In agreement with prior studies, our study indicated that males are 1.10–3.0‐fold more likely to have gastric cancer than females. In agreement with prior studies, our study found that the incidence of gastric cancer was 2.2–2.5 times higher in males than in females in the past decade. Such differences could be attributed to lifestyle (men are more likely to be influenced by cultural reasons and take up drinking and smoking than are women^14^ ), environmental or occupational exposures, and physiological differences.^15^, ^16^, ^17^ Moreover, the incidence and mortality rates decreased among both sexes in China. The decreasing trends of ASIR and ASMR were more pronounced in females. The control and prevention strategies for gastric cancer in males need to be strengthened.^18^, ^19^

Some risk factors, such as drinking, smoking, obesity, and unhealthy diet, increases the incidence levels of gastric cancer.^20^, ^21^ Excessive consumption of salty, pickled, preserved, and fried foods (which are rich in N‐nitroso compounds) in East Asian cultures, are proved to generate higher gastric cancer risk.^22^, ^23^ Besides, *H*. *pylori* (*Helicobacter pylori*) is a contributing factor for gastric cancer. The risk of developing gastric cancer in individuals infected with *H pylori* is at least two times higher than those who test negative.^24^

During the last decade, the incidence of gastric cancer has decreased steadily owing to the reduction in risk factors in China and other countries.^25^ The decline benefits from better food preservation associated with refrigeration during transport and storage. The increasing consumption of fresh fruit and vegetables also plays a vital role in bringing about the observed decline in the incidence of gastric cancer.^26^, ^27^ It is now clear that the decreasing trend in gastric cancer occurrence is parallel to the decline in *H*. *pylori* infection in both Eastern and Western populations.^28^, ^29^, ^30^, ^31^ Powerful measures for tobacco control decreased the incidence of gastric cancer.^32^, ^33^

The downward trend in mortality might be linked to several reasons. First, treatment for *H*. *pylori* eradication has a positive influence on the mortality of gastric cancer.^28^, ^34^, ^35^ In some prospective studies, vitamin supplementation (Vitamin C and E) or garlic was associated with the reduced mortality of gastric cancer.^28^, ^29^ Besides, an advanced understanding of gastric cancer helps clinicians to treat the disease and reduce mortality effectively. The selective screening programs for high‐risk populations carried out in the high‐risk regions significantly reduce the mortality rates in China.^36^, ^37^

Geographical variation in the incidence and mortality rates remains high across the world. High ASIR of gastric cancer have been reported in East Asia and High‐income Asia Pacific, whereas Africa and North America present low ASIR. Specific dietary patterns of the East Asian population mentioned above may account for the extremely high incidence within this region. Owing to effective screening strategies, the ASMR values in Japan and South Korea are much lower than those in other countries, despite the high ASIR.^38^, ^39^ One critical point that should be emphasized here is that the completeness of the data and the cancer registries in African countries might differ significantly from the countries with high HDI; therefore, these factors might introduce a bias in the results and interpretations. So the disease burden in these regions is possible to be underestimated.

DALYs is a comprehensive indicator which reflects the disease burden.^2^, ^4^ Because of the high incidence of gastric cancer and its large population, China is a major contributor to the global gastric cancer burden. From 1990 to 2019, the age‐standardized DALYs decreased more pronounced in women than in men in China. These data suggest that sex is an important factor affecting gastric cancer disease burden.^19^

On top of the above findings, there might be the following limitations in this paper that can be improved further. First, non‐cardia and cardia gastric cancer demonstrate remarkable characteristics in the epidemiology and risk factor profiles.^40^ Because of the incomplete cancer diagnostic and registration practices, subsite‐specific analysis of gastric epidemiology was not performed in this study. Second, we are not able to perform further analysis due to the lack of details regarding gastric cancer (such as classification, staging, and treatment received).

## 5 CONCLUSION

This study outlined the Chinese and global burdens of gastric cancer from 1990 to 2019. The ASIR, ASMR, and DALYs have decrease in China and the world. However, rates and temporal trends varied substantially by gender, age, socioeconomic status, and geography. The incident and mortality rates are relatively high in China. Moreover, there was a worrisome rise in the incidence of early‐onset gastric cancer in China. Specific strategies are needed to reduce the disease burden of gastric cancer.

## CONFLICT OF INTEREST

The authors declare that they have no competing interests.

## ETHICAL APPROVAL STATEMENT

Not applicable.

## Supporting information

Table S1

Table S2

Table S3

Table S4

Table S5

## Data Availability

Data and materials of this study are available from the corresponding author upon reasonable request.

## References

[1] Zhou M , Wang H , Zhu J , et al. Cause‐specific mortality for 240 causes in China during 1990–2013: a systematic subnational analysis for the Global Burden of Disease Study 2013. Lancet. 2016;387(10015):251‐272. 26510778 10.1016/S0140-6736(15)00551-6

[2] GBD 2017 Mortality Collaborators . Global, regional, and national age‐sex‐specific mortality and life expectancy, 1950‐2017: a systematic analysis for the Global Burden of Disease Study 2017. Lancet. 2018;392(10159):1684‐1735. 30496102 10.1016/S0140-6736(18)31891-9 PMC6227504

[3] GBD 2019 Diseases and Injuries Collaborators . Global burden of 369 diseases and injuries in 204 countries and territories, 1990‐2019: a systematic analysis for the Global Burden of Disease Study 2019. Lancet. 2020;396(10258):1204‐1222. 33069326 10.1016/S0140-6736(20)30925-9 PMC7567026

[4] Bray F , Ferlay J , Soerjomataram I , Siegel RL , Torre LA , Jemal A . Global cancer statistics 2018: GLOBOCAN estimates of incidence and mortality worldwide for 36 cancers in 185 countries. CA Cancer J Clin. 2018;68(6):394‐424. 30207593 10.3322/caac.21492

[5] Institute for health metrics and evaluation. 2017 http://ghdx.healthdata.org/gbd‐results‐tool.

[6] Institute for health metrics and evaluation. 2017 http://ghdx.healthdata.org/record/ihme‐data/gbd‐2017‐socio‐demographic‐index‐sdi‐1950%E2%80%932017.

[7] Ahmad OB , Boschi‐Pinto C , Lopez AD , Murray CJL , Lozano R . Mie Inoue. Age standardization of rates: a new WHO standard; 2001. https://www.who.int/healthinfo/paper31.pdf

[8] Wei W , Zeng H , Zheng R , et al. Cancer registration in China and its role in cancer prevention and control. Lancet Oncol. 2020;21(7):e342‐e349. 32615118 10.1016/S1470-2045(20)30073-5

[9] Arnold M , Park JY , Camargo MC , Lunet N , Forman D , Soerjomataram I . Is gastric cancer becoming a rare disease? A global assessment of predicted incidence trends to 2035. Gut. 2020;69(5):823‐829. 32001553 10.1136/gutjnl-2019-320234 PMC8520492

[10] Bergquist JR , Leiting JL , Habermann EB , et al. Early‐onset gastric cancer is a distinct disease with worrisome trends and oncogenic features. Surgery. 2019;166(4):547‐555. 31331685 10.1016/j.surg.2019.04.036

[11] Takatsu Y , Hiki N , Nunobe S , et al. Clinicopathological features of gastric cancer in young patients. Gastric Cancer. 2016;19(2):472‐478. 25752270 10.1007/s10120-015-0484-1

[12] Crew KD , Neugut AI . Epidemiology of gastric cancer. World J Gastroenterol. 2006;12(3):354‐362. 16489633 10.3748/wjg.v12.i3.354 PMC4066052

[13] Yang L . Incidence and mortality of gastric cancer in China. World J Gastroenterol. 2006;12(1):17‐20. 16440411 10.3748/wjg.v12.i1.17 PMC4077485

[14] Kendler KS , Thornton LM , Pedersen NL . Tobacco consumption in Swedish twins reared apart and reared together. Arch Gen Psychiatry. 2000;57(9):886‐892. 10986552 10.1001/archpsyc.57.9.886

[15] Camargo MC , Goto Y , Zabaleta J , Morgan DR , Correa P , Rabkin CS . Sex hormones, hormonal interventions, and gastric cancer risk: a meta‐analysis. Cancer Epidemiol Biomarkers Prev. 2012;21(1):20‐38. 22028402 10.1158/1055-9965.EPI-11-0834 PMC3315355

[16] González CA , Agudo A . Carcinogenesis, prevention and early detection of gastric cancer: where we are and where we should go. Int J Cancer. 2012;130(4):745‐753. 21918974 10.1002/ijc.26430

[17] Derakhshan MH , Liptrot S , Paul J , Brown IL , Morrison D , McColl KEL . Oesophageal and gastric intestinal‐type adenocarcinomas show the same male predominance due to a 17 year delayed development in females. Gut. 2009;58(1):16‐23. 18838486 10.1136/gut.2008.161331

[18] Pérez‐Rodríguez M , Partida‐Rodríguez O , Camorlinga‐Ponce M , et al. Polymorphisms in HLA‐DQ genes, together with age, sex, and Helicobacter pylori infection, as potential biomarkers for the early diagnosis of gastric cancer. Helicobacter. 2017;22(1):e12326. 10.1111/hel.12326 27334226

[19] Song M , Kang D , Yang JJ , et al. Age and sex interactions in gastric cancer incidence and mortality trends in Korea. Gastric Cancer. 2015;18(3):580‐589. 25091081 10.1007/s10120-014-0411-x

[20] Karimi P , Islami F , Anandasabapathy S , Freedman ND , Kamangar F . Gastric cancer: descriptive epidemiology, risk factors, screening, and prevention. Cancer Epidemiol Biomark Prev. 2014;23(5):700‐713. 10.1158/1055-9965.EPI-13-1057 PMC4019373 24618998

[21] Fang X , Wei J , He X , et al. Landscape of dietary factors associated with risk of gastric cancer: A systematic review and dose‐response meta‐analysis of prospective cohort studies. Eur J Cancer. 2015;51(18):2820‐2832. 26589974 10.1016/j.ejca.2015.09.010

[22] Shikata K , Kiyohara Y , Kubo M , et al. A prospective study of dietary salt intake and gastric cancer incidence in a defined Japanese population: the Hisayama study. Int J Cancer. 2006;119(1):196‐201. 16450397 10.1002/ijc.21822

[23] Leung WK , Wu M‐S , Kakugawa Y , et al. Screening for gastric cancer in Asia: current evidence and practice. Lancet Oncol. 2008;9(3):279‐287. 18308253 10.1016/S1470-2045(08)70072-X

[24] Eid R , Moss SF . Helicobacter pylori infection and the development of gastric cancer. N Engl J Med. 2002;346(1):65‐67. 11799959

[25] Peleteiro B , Padrão P , Castro C , Ferro A , Morais S , Lunet N . Worldwide burden of gastric cancer in 2012 that could have been prevented by increasing fruit and vegetable intake and predictions for 2025. Br J Nutr. 2016;115(5):851‐859. 26794617 10.1017/S000711451500522X

[26] Kobayashi M , Tsubono Y , Sasazuki S , Sasaki S , Tsugane S . Vegetables, fruit and risk of gastric cancer in Japan: a 10‐year follow‐up of the JPHC Study Cohort I. Int J Cancer. 2002;102(1):39‐44. 12353232 10.1002/ijc.10659

[27] Wang T , Cai H , Sasazuki S , et al. Fruit and vegetable consumption, Helicobacter pylori antibodies, and gastric cancer risk: A pooled analysis of prospective studies in China, Japan, and Korea. Int J Cancer. 2017;140(3):591‐599. 27759938 10.1002/ijc.30477 PMC5531280

[28] Ma J‐L , Zhang L , Brown LM , et al. Fifteen‐year effects of Helicobacter pylori, garlic, and vitamin treatments on gastric cancer incidence and mortality. J Natl Cancer Inst. 2012;104(6):488‐492. 22271764 10.1093/jnci/djs003 PMC3309129

[29] Li W‐Q , Zhang J‐Y , Ma J‐L , et al. Effects of treatment and vitamin and garlic supplementation on gastric cancer incidence and mortality: follow‐up of a randomized intervention trial. BMJ. 2019;366:l5016. 31511230 10.1136/bmj.l5016 PMC6737461

[30] Doorakkers E , Lagergren J , Engstrand L , Brusselaers N . eradication treatment and the risk of gastric adenocarcinoma in a Western population. Gut. 2018;67(12):2092‐2096. 29382776 10.1136/gutjnl-2017-315363

[31] Uemura N , Okamoto S , Yamamoto S , et al. Helicobacter pylori infection and the development of gastric cancer. N Engl J Med. 2001;345(11):784‐789. 11556297 10.1056/NEJMoa001999

[32] Center CDCaP . Tobacco survey report in adult China. 2018; 2018. http://www.chinacdc.cn/yw_9324/201905/t20190530_202932.html.

[33] IARC Monogr Eval Carcinog Risks Hum . Tobacco smoke and involuntary smoking. 2004;83. PMC4781536 15285078

[34] Tsuda M , Asaka M , Kato M , et al. Effect on Helicobacter pylori eradication therapy against gastric cancer in Japan. Helicobacter. 2017;22(5):e12415. 10.1111/hel.12415 PMC5655764 28771894

[35] Li W‐Q , Ma J‐L , Zhang L , et al. Effects of Helicobacter pylori treatment on gastric cancer incidence and mortality in subgroups. J Natl Cancer Inst. 2014;106(7):dju116. 24925350 10.1093/jnci/dju116 PMC4067110

[36] Zhang X , Li M , Chen S , et al. Endoscopic screening in Asian Countries is associated with reduced gastric cancer mortality: a meta‐analysis and systematic review. Gastroenterology. 2018;155(2):347‐54.e9. 29723507 10.1053/j.gastro.2018.04.026

[37] Leung WK , Ho HJ , Lin J‐T , Wu M‐S , Wu C‐Y . Prior gastroscopy and mortality in patients with gastric cancer: a matched retrospective cohort study. Gastrointest Endosc. 2018;87(1):119‐127.e3. 28648576 10.1016/j.gie.2017.06.013

[38] Hamashima C , Shabana M , Okada K , Okamoto M , Osaki Y . Mortality reduction from gastric cancer by endoscopic and radiographic screening. Cancer Sci. 2015;106(12):1744‐1749. 26432528 10.1111/cas.12829 PMC4714659

[39] Suzuki H , Gotoda T , Sasako M , Saito D . Detection of early gastric cancer: misunderstanding the role of mass screening. Gastric Cancer. 2006;9(4):315‐319. 17235635 10.1007/s10120-006-0399-y

[40] Colquhoun A , Arnold M , Ferlay J , Goodman KJ , Forman D , Soerjomataram I . Global patterns of cardia and non‐cardia gastric cancer incidence in 2012. Gut. 2015;64(12):1881‐1888. 25748648 10.1136/gutjnl-2014-308915

