# Supplementary material for: Chinese and global burdens of gastric cancer from 1990 to 2019
Source: Cancer Med. 2021 May 1;10(10):3461–73. doi: 10.1002/cam4.3892 (PMC8124120; doi:10.1002/cam4.3892)
Supplement: Supplementary file 1 — Table S1 [file CAM4-10-3461-s004.docx]

**Table S1. The change trends of gastric cancer incidence, mortality and DALYs among genders in China from 1990 to 2019**

| **Characteristics** | 1990-2019 increase (%) | | |  | eAPC (95%CI) | | |
| --- | --- | --- | --- | --- | --- | --- | --- |
|  | Incidence cases | Deaths | DALYs |  | ASIR | ASMR | Age-standardized DALY |
| Both genders | 0.93(0.56,1.40) | 0.38(0.12,0.70) | 0.32(-0.01,0.76) |  | -0.18(-0.33,0.01) | -0.42(-0.53,-0.30) | -0.47(-0.57,-0.35) |
| Males | 1.17(0.65,1.88) | 0.51(0.14,1.02) | 0.32(-0.01,0.76) |  | -0.07(-0.29,0.23) | -0.35(-0.50,-0.16) | -0.40(-0.55,-0.21) |
| Females | 0.47(0.12,0.91) | 0.13(-0.12,0.48) | -0.05(-0.27,0.26) |  | -0.38(-0.53,-0.20) | -0.53(-0.63,-0.40) | -0.58(-0.67,-0.44) |

ASIR, age-standardized incidence rate; ASMR, age-standardized mortality rate; DALY, disability-adjusted life year; eAPC, estimated annual percentage change; CI, confidence interval
