# Supplementary material for: Chinese and global burdens of gastric cancer from 1990 to 2019
Source: Cancer Med. 2021 May 1;10(10):3461–73. doi: 10.1002/cam4.3892 (PMC8124120; doi:10.1002/cam4.3892)
Supplement: Supplementary file 2 — Table S2 [file CAM4-10-3461-s005.docx]

**Table S2. The ASRs and variations of TBL cancer from 1990 to 2017 among countries and territories.**

| **Characteristics** | **ASIR (per 100,000 persons)** | | | | |  | **ASMR (per 100,000 persons)** | | | | |  | **DALYS (per 100,000 persons)** | | | | |
| --- | --- | --- | --- | --- | --- | --- | --- | --- | --- | --- | --- | --- | --- | --- | --- | --- | --- |
|  | 1990 (95%CI) | | 2019 (95%CI) | | eAPC  (95%CI) |  | 1990 (95%CI) | | 2019 (95%CI) | | eAPC  (95%CI) |  | 1990 (95%CI) | | 2019 (95%CI) | | eAPC  (95%CI) |
|  | Both | Male/ female  ratio | Both | Male/ female  ratio |  |  | Both | Male/ female  ratio | Both | Male/ female  ratio |  |  | Both | Male/ female  ratio | Both | Male/ female  ratio |  |
| Afghanistan | 30.39  (21.63,38.63) | 1.34 | 27.69  (19.94,34.87) | 1.26 | -0.09  (-0.31,0.16) |  | 31.99  (23.12,40.19) | 1.38 | 29.30  (21.25,36.52) | 1.30 | -0.08  (-0.30,0.16) |  | 828.00  (546.45,1071.09) | 1.18 | 728.70  (505.26,939.40) | 1.14 | -0.12  (-0.34,0.16) |
| Albania | 15.72  (14.73,16.73) | 2.20 | 11.60  (8.72,15.35) | 2.58 | -0.26  (-0.45,-0.03) |  | 16.12  (15.09,17.21) | 2.23 | 10.68  (8.05,14.07) | 2.58 | -0.34  (-0.50,-0.13) |  | 386.99  (363.90,409.18) | 2.10 | 251.64  (186.40,335.21) | 2.69 | -0.35  (-0.52,-0.14) |
| Algeria | 8.75  (7.07,10.56) | 1.74 | 4.98  (4.06,6.09) | 1.56 | -0.43  (-0.57,-0.28) |  | 9.62  (7.87,11.59) | 1.73 | 5.10  (4.17,6.18) | 1.53 | -0.47  (-0.60,-0.33) |  | 197.79  (157.88,240.98) | 1.65 | 102.61  (82.49,126.74) | 1.52 | -0.48  (-0.62,-0.33) |
| American Samoa | 18.86  (16.47,21.49) | 2.05 | 15.46  (13.04,18.30) | 1.68 | -0.18  (-0.33,0.01) |  | 19.81  (17.44,22.46) | 1.92 | 15.79  (13.39,18.55) | 1.58 | -0.20  (-0.35,-0.03) |  | 464.43  (402.78,534.04) | 2.27 | 368.26  (305.35,447.79) | 1.83 | -0.21  (-0.37,0.00) |
| Andorra | 14.91  (11.61,20.00) | 1.69 | 12.10  (9.15,15.74) | 1.52 | -0.19  (-0.44,0.11) |  | 12.53  (9.85,16.63) | 1.63 | 8.30  (6.34,10.66) | 1.43 | -0.34  (-0.54,-0.10) |  | 252.11  (194.46,341.94) | 1.67 | 165.92  (125.12,216.98) | 1.45 | -0.34  (-0.56,-0.08) |
| Angola | 13.48  (10.52,17.03) | 2.15 | 8.39  (6.79,10.61) | 2.10 | -0.38  (-0.53,-0.18) |  | 14.34  (11.22,17.92) | 2.18 | 8.96  (7.31,11.24) | 2.09 | -0.38  (-0.52,-0.18) |  | 364.51  (279.71,466.12) | 2.11 | 211.90  (166.49,269.74) | 2.09 | -0.42  (-0.57,-0.22) |
| Antigua and Barbuda | 16.19  (14.80,17.65) | 3.34 | 11.37  (9.74,13.05) | 1.91 | -0.30  (-0.41,-0.18) |  | 16.47  (15.03,17.93) | 3.31 | 11.05  (9.48,12.67) | 1.88 | -0.33  (-0.43,-0.22) |  | 371.09  (338.60,405.93) | 3.31 | 226.39  (192.52,263.60) | 1.92 | -0.39  (-0.49,-0.28) |
| Argentina | 14.12  (13.51,14.69) | 2.40 | 9.89  (7.87,12.44) | 2.34 | -0.30  (-0.44,-0.12) |  | 14.62  (13.97,15.23) | 2.35 | 9.44  (8.78,10.15) | 2.29 | -0.35  (-0.40,-0.30) |  | 327.49  (314.28,340.35) | 2.46 | 212.75  (199.45,227.14) | 2.33 | -0.35  (-0.40,-0.30) |
| Armenia | 22.88  (21.93,23.90) | 2.21 | 13.40  (11.19,15.74) | 2.25 | -0.41  (-0.51,-0.31) |  | 22.79  (21.84,23.79) | 2.20 | 13.01  (10.90,15.23) | 2.25 | -0.43  (-0.52,-0.33) |  | 606.17  (580.73,632.63) | 2.28 | 304.52  (252.94,360.31) | 2.37 | -0.50  (-0.58,-0.40) |
| Australia | 10.02  (9.53,10.47) | 2.27 | 7.01  (5.46,8.81) | 2.10 | -0.30  (-0.45,-0.12) |  | 7.30  (6.92,7.58) | 2.12 | 3.89  (3.51,4.23) | 1.92 | -0.47  (-0.50,-0.42) |  | 154.15  (148.26,159.38) | 2.11 | 81.42  (74.99,87.73) | 1.92 | -0.47  (-0.51,-0.43) |
| Austria | 20.59  (19.65,21.46) | 2.09 | 8.43  (6.83,10.22) | 1.93 | -0.59  (-0.67,-0.50) |  | 17.29  (16.50,17.89) | 2.05 | 5.42  (4.96,5.87) | 1.81 | -0.69  (-0.71,-0.66) |  | 372.84  (359.21,384.94) | 2.08 | 110.44  (102.62,118.38) | 1.80 | -0.70  (-0.72,-0.68) |
| Azerbaijan | 29.78  (27.52,32.12) | 2.57 | 21.72  (18.14,26.38) | 2.19 | -0.27  (-0.40,-0.10) |  | 30.24  (28.01,32.60) | 2.54 | 22.48  (18.75,27.33) | 2.15 | -0.26  (-0.39,-0.08) |  | 801.29  (740.06,866.47) | 2.66 | 502.49  (418.68,608.42) | 2.32 | -0.37  (-0.49,-0.22) |
| Bahamas | 14.05  (12.81,15.40) | 1.96 | 9.35  (7.61,11.37) | 1.71 | -0.33  (-0.47,-0.17) |  | 14.26  (13.00,15.63) | 1.95 | 9.16  (7.51,11.14) | 1.68 | -0.36  (-0.48,-0.21) |  | 341.20  (311.31,372.16) | 1.94 | 213.41  (172.01,263.33) | 1.76 | -0.37  (-0.51,-0.21) |
| Bahrain | 13.65  (11.75,15.55) | 1.47 | 6.46  (5.24,7.82) | 1.29 | -0.53  (-0.62,-0.40) |  | 14.71  (12.67,16.67) | 1.49 | 6.29  (5.13,7.58) | 1.29 | -0.57  (-0.66,-0.46) |  | 297.45  (255.29,340.92) | 1.38 | 114.12  (91.68,139.96) | 1.23 | -0.62  (-0.70,-0.50) |
| Bangladesh | 10.99  (9.21,12.65) | 1.72 | 6.30  (4.83,8.27) | 1.27 | -0.43  (-0.56,-0.23) |  | 11.56  (9.71,13.33) | 1.73 | 6.58  (5.07,8.60) | 1.27 | -0.43  (-0.56,-0.24) |  | 291.65  (241.50,339.03) | 1.62 | 155.42  (117.65,208.64) | 1.21 | -0.47  (-0.60,-0.27) |
| Barbados | 16.02  (14.79,17.23) | 2.18 | 10.91  (9.00,12.84) | 1.68 | -0.32  (-0.44,-0.19) |  | 16.40  (15.04,17.66) | 2.13 | 10.41  (8.65,12.23) | 1.64 | -0.37  (-0.48,-0.24) |  | 357.75  (332.33,384.19) | 2.22 | 219.65  (180.02,263.99) | 1.72 | -0.39  (-0.50,-0.26) |
| Belarus | 35.40  (34.10,36.66) | 2.18 | 17.86  (14.13,22.99) | 1.96 | -0.50  (-0.60,-0.35) |  | 33.03  (31.81,34.16) | 2.34 | 14.10  (11.16,17.92) | 2.39 | -0.57  (-0.66,-0.45) |  | 888.02  (857.04,918.78) | 2.36 | 349.91  (273.73,454.59) | 2.36 | -0.61  (-0.69,-0.49) |
| Belgium | 13.06  (12.30,13.76) | 1.98 | 7.05  (5.59,8.85) | 2.17 | -0.46  (-0.57,-0.32) |  | 11.99  (11.20,12.61) | 1.91 | 5.12  (4.67,5.54) | 2.01 | -0.57  (-0.60,-0.54) |  | 227.77  (217.39,237.54) | 2.01 | 100.79  (93.24,107.60) | 2.13 | -0.56  (-0.59,-0.52) |
| Belize | 12.20  (10.92,13.49) | 1.42 | 10.12  (8.74,11.66) | 1.70 | -0.17  (-0.31,-0.02) |  | 12.92  (11.62,14.24) | 1.38 | 10.17  (8.81,11.72) | 1.66 | -0.21  (-0.34,-0.07) |  | 283.96  (255.54,313.00) | 1.44 | 238.11  (204.90,273.93) | 1.74 | -0.16  (-0.30,0.00) |
| Benin | 15.86  (13.72,18.24) | 1.81 | 12.37  (9.96,15.43) | 1.62 | -0.22  (-0.38,-0.04) |  | 17.38  (15.10,19.89) | 1.83 | 13.66  (11.09,16.75) | 1.62 | -0.21  (-0.37,-0.03) |  | 383.81  (332.26,445.69) | 1.81 | 285.52  (221.79,367.23) | 1.63 | -0.26  (-0.42,-0.06) |
| Bermuda | 13.11  (12.00,14.27) | 3.21 | 6.70  (5.59,8.00) | 3.37 | -0.49  (-0.57,-0.38) |  | 12.81  (11.72,13.99) | 3.09 | 5.07  (4.24,6.01) | 3.20 | -0.60  (-0.67,-0.52) |  | 275.87  (252.63,299.57) | 3.34 | 103.49  (86.70,124.90) | 3.52 | -0.62  (-0.69,-0.54) |
| Bhutan | 9.29  (6.60,12.23) | 1.24 | 7.96  (5.82,10.37) | 1.11 | -0.14  (-0.37,0.20) |  | 9.81  (7.11,12.82) | 1.27 | 8.41  (6.21,10.85) | 1.12 | -0.14  (-0.36,0.18) |  | 248.62  (169.28,333.72) | 1.15 | 188.24  (134.07,253.25) | 1.06 | -0.24  (-0.46,0.10) |
| Bolivia  (Plurinational State of) | 45.31  (37.02,52.85) | 1.26 | 34.02  (26.85,42.02) | 1.21 | -0.25  (-0.41,-0.03) |  | 49.08  (40.31,57.21) | 1.27 | 36.11  (28.77,44.26) | 1.16 | -0.26  (-0.42,-0.06) |  | 1116.97  (912.10,1314.83) | 1.24 | 749.11  (572.68,946.36) | 1.16 | -0.33  (-0.49,-0.12) |
| Bosnia and Herzegovina | 13.64  (12.89,14.37) | 2.16 | 10.78  (8.52,13.61) | 2.11 | -0.21  (-0.37,0.00) |  | 13.89  (13.14,14.67) | 2.15 | 10.31  (8.24,12.97) | 2.14 | -0.26  (-0.41,-0.06) |  | 335.68  (318.19,354.23) | 2.16 | 234.58  (183.90,298.78) | 2.08 | -0.30  (-0.45,-0.10) |
| Botswana | 11.13  (8.53,14.13) | 2.01 | 8.66  (6.60,10.98) | 1.96 | -0.22  (-0.44,0.06) |  | 11.92  (9.21,14.99) | 1.96 | 9.04  (6.99,11.42) | 1.91 | -0.24  (-0.45,0.02) |  | 279.26  (209.62,361.27) | 2.12 | 207.07  (151.84,267.87) | 2.05 | -0.26  (-0.49,0.04) |
| Brazil | 18.27  (17.42,18.88) | 2.22 | 10.23  (9.60,10.71) | 2.23 | -0.44  (-0.47,-0.41) |  | 19.11  (18.09,19.80) | 2.16 | 9.87  (9.14,10.37) | 2.20 | -0.48  (-0.51,-0.46) |  | 437.07  (419.36,450.97) | 2.29 | 226.31  (214.84,236.08) | 2.20 | -0.48  (-0.51,-0.45) |
| Brunei Darussalam | 35.56  (31.50,40.08) | 1.92 | 17.31  (15.13,19.53) | 1.83 | -0.51  (-0.60,-0.42) |  | 32.75  (29.02,36.72) | 2.01 | 14.11  (12.38,15.85) | 1.92 | -0.57  (-0.64,-0.49) |  | 748.04  (658.09,842.75) | 1.63 | 299.66  (261.94,338.01) | 1.60 | -0.60  (-0.67,-0.52) |
| Bulgaria | 19.33  (18.29,20.35) | 1.82 | 11.59  (9.24,14.50) | 2.20 | -0.40  (-0.53,-0.25) |  | 19.44  (18.40,20.45) | 1.78 | 10.81  (8.65,13.46) | 2.11 | -0.44  (-0.56,-0.31) |  | 464.59  (438.82,489.70) | 1.83 | 259.77  (204.41,325.43) | 2.10 | -0.44  (-0.56,-0.29) |
| Burkina Faso | 16.74  (13.78,19.82) | 1.72 | 14.01  (11.52,16.83) | 1.65 | -0.16  (-0.32,0.02) |  | 18.34  (15.16,21.64) | 1.72 | 15.38  (12.72,18.31) | 1.65 | -0.16  (-0.32,0.02) |  | 402.86  (325.02,483.50) | 1.71 | 328.13  (265.63,399.54) | 1.68 | -0.19  (-0.35,0.01) |
| Burundi | 12.71  (10.27,15.47) | 2.06 | 8.43  (6.59,10.70) | 1.71 | -0.34  (-0.49,-0.13) |  | 13.46  (11.00,16.21) | 2.11 | 8.99  (7.12,11.37) | 1.74 | -0.33  (-0.49,-0.12) |  | 348.23  (277.86,429.54) | 1.97 | 220.98  (170.01,284.89) | 1.67 | -0.37  (-0.52,-0.14) |
| Cabo Verde | 30.19  (26.95,33.49) | 1.54 | 23.79  (20.66,26.99) | 2.86 | -0.21  (-0.33,-0.07) |  | 32.69  (29.24,36.37) | 1.49 | 25.65  (22.46,28.98) | 2.74 | -0.22  (-0.33,-0.08) |  | 717.42  (638.17,800.92) | 1.68 | 502.33  (428.37,581.95) | 3.22 | -0.30  (-0.42,-0.16) |
| Cambodia | 14.80  (11.61,17.85) | 1.70 | 8.88  (7.13,10.59) | 1.81 | -0.40  (-0.54,-0.22) |  | 15.64  (12.30,18.85) | 1.75 | 9.35  (7.53,11.05) | 1.82 | -0.40  (-0.54,-0.23) |  | 395.99  (305.11,481.23) | 1.62 | 213.80  (169.38,261.62) | 1.77 | -0.46  (-0.60,-0.29) |
| Cameroon | 15.73  (13.21,18.38) | 1.61 | 13.01  (10.12,16.59) | 1.73 | -0.17  (-0.37,0.08) |  | 17.24  (14.52,20.25) | 1.63 | 14.24  (11.19,17.99) | 1.71 | -0.17  (-0.36,0.07) |  | 381.38  (318.10,452.80) | 1.59 | 301.55  (228.38,394.67) | 1.76 | -0.21  (-0.41,0.06) |
| Canada | 10.52  (9.95,11.00) | 2.39 | 7.99  (6.25,10.04) | 2.42 | -0.24  (-0.41,-0.04) |  | 7.60  (7.20,7.91) | 2.07 | 4.40  (4.00,4.76) | 1.90 | -0.42  (-0.46,-0.38) |  | 160.94  (154.75,166.36) | 2.07 | 90.44  (84.15,97.31) | 1.88 | -0.44  (-0.48,-0.39) |
| Central African Republic | 16.19  (13.28,19.41) | 2.32 | 11.79  (8.93,15.28) | 2.29 | -0.27  (-0.44,-0.06) |  | 17.16  (14.13,20.49) | 2.36 | 12.46  (9.46,16.00) | 2.29 | -0.27  (-0.44,-0.07) |  | 454.13  (369.00,547.01) | 2.38 | 326.77  (242.95,429.18) | 2.35 | -0.28  (-0.46,-0.06) |
| Chad | 15.10  (12.78,17.72) | 1.58 | 14.88  (11.80,18.40) | 1.43 | -0.01  (-0.22,0.23) |  | 16.50  (13.94,19.37) | 1.60 | 16.38  (13.04,20.17) | 1.44 | -0.01  (-0.21,0.22) |  | 369.36  (311.01,434.59) | 1.56 | 351.08  (274.02,445.44) | 1.44 | -0.05  (-0.26,0.21) |
| Chile | 33.57  (32.05,34.90) | 2.34 | 19.87  (15.80,24.84) | 2.45 | -0.41  (-0.53,-0.25) |  | 34.05  (32.32,35.44) | 2.21 | 17.63  (16.17,18.99) | 2.35 | -0.48  (-0.52,-0.44) |  | 734.85  (705.57,763.87) | 2.47 | 360.03  (335.42,383.76) | 2.47 | -0.51  (-0.55,-0.47) |
| China | 37.56  (33.08,42.27) | 2.00 | 30.64  (25.82,36.15) | 3.00 | -0.18  (-0.33,0.01) |  | 37.73  (33.20,42.39) | 1.96 | 21.72  (18.31,25.31) | 2.72 | -0.42  (-0.53,-0.30) |  | 905.54  (791.75,1024.49) | 1.95 | 481.15  (403.20,567.36) | 2.75 | -0.47  (-0.57,-0.35) |
| Colombia | 29.37  (27.91,30.51) | 1.39 | 14.99  (11.67,19.02) | 1.58 | -0.49  (-0.61,-0.35) |  | 30.09  (28.44,31.36) | 1.39 | 12.73  (9.91,16.09) | 1.68 | -0.58  (-0.67,-0.46) |  | 670.90  (645.50,694.85) | 1.48 | 294.24  (225.42,375.62) | 1.70 | -0.56  (-0.67,-0.43) |
| Comoros | 9.32  (5.64,12.28) | 1.66 | 6.67  (5.30,8.41) | 1.41 | -0.28  (-0.46,0.23) |  | 10.02  (6.21,13.08) | 1.72 | 7.16  (5.76,8.95) | 1.44 | -0.29  (-0.46,0.16) |  | 241.44  (130.51,326.72) | 1.57 | 168.38  (127.17,216.31) | 1.34 | -0.30  (-0.50,0.36) |
| Congo | 14.45  (11.91,17.37) | 1.98 | 8.09  (6.57,10.02) | 1.57 | -0.44  (-0.56,-0.28) |  | 15.34  (12.65,18.30) | 2.00 | 8.67  (7.14,10.66) | 1.58 | -0.43  (-0.55,-0.28) |  | 391.65  (319.66,474.49) | 2.00 | 203.09  (159.52,260.10) | 1.53 | -0.48  (-0.61,-0.32) |
| Cook Islands | 11.07  (9.58,12.56) | 2.17 | 7.48  (6.20,9.09) | 2.62 | -0.32  (-0.46,-0.14) |  | 11.09  (9.63,12.54) | 2.05 | 6.75  (5.66,8.16) | 2.33 | -0.39  (-0.51,-0.23) |  | 261.93  (223.31,303.99) | 2.28 | 155.64  (125.20,193.75) | 2.93 | -0.41  (-0.55,-0.21) |
| Costa Rica | 39.42  (36.98,41.48) | 2.03 | 23.26  (18.21,29.36) | 1.85 | -0.41  (-0.54,-0.25) |  | 37.47  (34.82,39.51) | 2.13 | 19.02  (14.80,23.93) | 2.00 | -0.49  (-0.60,-0.36) |  | 817.37  (772.97,857.52) | 2.13 | 412.26  (319.83,525.18) | 2.01 | -0.50  (-0.61,-0.35) |
| Croatia | 24.23  (22.62,25.77) | 2.19 | 12.24  (9.73,15.18) | 2.01 | -0.49  (-0.60,-0.37) |  | 22.16  (20.81,23.55) | 2.29 | 9.38  (7.50,11.57) | 2.23 | -0.58  (-0.66,-0.47) |  | 503.27  (472.15,534.29) | 2.41 | 200.73  (157.83,251.72) | 2.28 | -0.60  (-0.69,-0.49) |
| Cuba | 7.85  (7.45,8.21) | 1.82 | 6.61  (5.38,8.08) | 1.84 | -0.16  (-0.31,0.04) |  | 7.71  (7.29,8.07) | 1.80 | 5.67  (4.63,6.89) | 1.79 | -0.26  (-0.40,-0.10) |  | 171.83  (164.89,179.55) | 1.79 | 125.50  (100.85,154.52) | 1.89 | -0.27  (-0.41,-0.10) |
| Cyprus | 9.00  (7.81,10.01) | 1.35 | 9.00  (7.85,10.27) | 1.58 | 0.00  (-0.16,0.24) |  | 9.09  (7.83,10.12) | 1.28 | 6.66  (5.87,7.60) | 1.41 | -0.27  (-0.38,-0.11) |  | 176.71  (154.73,196.74) | 1.43 | 129.05  (113.02,147.75) | 1.57 | -0.27  (-0.39,-0.11) |
| Czechia | 18.31  (17.61,19.00) | 2.08 | 8.00  (6.50,9.71) | 1.88 | -0.56  (-0.65,-0.47) |  | 17.56  (16.81,18.25) | 2.09 | 6.08  (4.98,7.33) | 1.94 | -0.65  (-0.72,-0.58) |  | 384.83  (371.85,399.10) | 2.14 | 131.61  (106.28,160.11) | 1.89 | -0.66  (-0.72,-0.58) |
| C’te d'Ivoire | 17.62  (14.59,20.76) | 2.02 | 13.33  (10.81,16.37) | 1.76 | -0.24  (-0.41,-0.05) |  | 19.22  (15.94,22.55) | 2.01 | 14.67  (12.01,17.84) | 1.75 | -0.24  (-0.39,-0.05) |  | 429.16  (349.18,511.06) | 2.05 | 307.68  (240.47,384.51) | 1.80 | -0.28  (-0.46,-0.08) |
| Democratic People's Republic of Korea | 31.13  (24.45,38.90) | 2.16 | 23.40  (18.56,28.92) | 2.44 | -0.25  (-0.42,-0.02) |  | 30.80  (24.09,38.31) | 2.12 | 22.49  (17.92,27.59) | 2.41 | -0.27  (-0.43,-0.06) |  | 812.22  (611.76,1039.17) | 2.12 | 577.22  (448.98,731.95) | 2.38 | -0.29  (-0.47,-0.04) |
| Democratic Republic of the Congo | 10.99  (8.99,13.31) | 1.88 | 7.62  (5.91,9.63) | 1.87 | -0.31  (-0.48,-0.10) |  | 11.80  (9.70,14.16) | 1.91 | 8.11  (6.32,10.24) | 1.88 | -0.31  (-0.48,-0.12) |  | 284.18  (228.34,349.99) | 1.85 | 195.53  (149.08,250.48) | 1.86 | -0.31  (-0.49,-0.10) |
| Denmark | 11.05  (10.55,11.49) | 1.98 | 6.16  (4.77,7.80) | 2.28 | -0.44  (-0.56,-0.29) |  | 9.45  (9.02,9.80) | 1.86 | 4.33  (3.93,4.72) | 1.94 | -0.54  (-0.58,-0.50) |  | 211.18  (203.00,219.00) | 1.85 | 91.19  (83.19,99.04) | 1.96 | -0.57  (-0.61,-0.53) |
| Djibouti | 9.39  (7.10,12.34) | 1.68 | 7.60  (5.84,10.13) | 1.63 | -0.19  (-0.39,0.11) |  | 10.07  (7.67,13.13) | 1.72 | 8.17  (6.37,10.74) | 1.64 | -0.19  (-0.38,0.11) |  | 243.10  (178.31,325.33) | 1.62 | 191.10  (140.34,265.09) | 1.60 | -0.21  (-0.43,0.12) |
| Dominica | 27.66  (24.65,30.73) | 1.69 | 19.79  (16.20,24.01) | 1.53 | -0.28  (-0.43,-0.12) |  | 28.84  (25.76,31.93) | 1.66 | 20.49  (16.91,24.66) | 1.49 | -0.29  (-0.42,-0.13) |  | 642.08  (562.54,727.09) | 1.70 | 441.78  (356.02,545.03) | 1.57 | -0.31  (-0.46,-0.14) |
| Dominican Republic | 7.20  (6.30,8.13) | 1.55 | 8.64  (6.54,11.38) | 1.96 | 0.20  (-0.10,0.59) |  | 7.70  (6.76,8.69) | 1.52 | 8.70  (6.68,11.31) | 1.89 | 0.13  (-0.15,0.47) |  | 181.48  (158.57,205.97) | 1.63 | 206.98  (153.25,276.83) | 2.05 | 0.14  (-0.17,0.54) |
| Ecuador | 30.02  (28.17,31.70) | 1.27 | 22.30  (17.83,28.19) | 1.58 | -0.26  (-0.41,-0.07) |  | 32.39  (30.17,34.24) | 1.20 | 21.86  (17.57,27.51) | 1.44 | -0.33  (-0.46,-0.16) |  | 701.62  (667.61,738.39) | 1.33 | 451.51  (354.46,580.55) | 1.46 | -0.36  (-0.49,-0.18) |
| Egypt | 5.63  (5.12,6.07) | 1.38 | 5.01  (3.82,6.52) | 1.27 | -0.11  (-0.32,0.18) |  | 6.11  (5.55,6.59) | 1.36 | 5.21  (4.00,6.76) | 1.21 | -0.15  (-0.35,0.12) |  | 134.70  (123.29,146.05) | 1.43 | 111.45  (83.66,147.44) | 1.39 | -0.17  (-0.39,0.12) |
| El Salvador | 16.35  (15.22,17.40) | 1.13 | 17.47  (13.38,22.37) | 1.46 | 0.07  (-0.19,0.38) |  | 16.89  (15.56,18.01) | 1.14 | 16.24  (12.51,20.62) | 1.51 | -0.04  (-0.27,0.23) |  | 407.04  (381.84,429.99) | 1.14 | 368.04  (277.49,479.94) | 1.59 | -0.10  (-0.32,0.19) |
| Equatorial Guinea | 13.50  (10.23,16.96) | 2.39 | 6.09  (4.48,8.40) | 1.72 | -0.55  (-0.69,-0.34) |  | 14.30  (10.90,18.02) | 2.44 | 6.58  (4.92,9.00) | 1.71 | -0.54  (-0.67,-0.34) |  | 370.72  (272.53,473.04) | 2.41 | 143.76  (101.47,203.63) | 1.72 | -0.61  (-0.74,-0.41) |
| Eritrea | 11.64  (8.87,14.96) | 2.18 | 9.93  (7.57,12.66) | 1.68 | -0.15  (-0.36,0.14) |  | 12.16  (9.24,15.68) | 2.21 | 10.45  (8.07,13.25) | 1.67 | -0.14  (-0.35,0.14) |  | 328.79  (248.45,420.12) | 2.13 | 264.36  (197.37,346.05) | 1.67 | -0.20  (-0.40,0.09) |
| Estonia | 29.01  (27.67,30.10) | 1.97 | 15.83  (12.34,20.28) | 1.55 | -0.45  (-0.58,-0.30) |  | 26.89  (25.71,27.93) | 2.14 | 11.38  (8.93,14.48) | 1.94 | -0.58  (-0.67,-0.46) |  | 681.26  (651.91,709.43) | 2.16 | 268.42  (208.05,346.14) | 1.94 | -0.61  (-0.70,-0.49) |
| Eswatini | 10.35  (8.27,12.53) | 2.08 | 8.57  (6.54,11.12) | 2.38 | -0.17  (-0.39,0.15) |  | 11.11  (8.91,13.44) | 2.04 | 9.15  (6.98,11.75) | 2.28 | -0.18  (-0.39,0.13) |  | 259.91  (203.98,320.02) | 2.15 | 217.07  (159.79,287.93) | 2.65 | -0.16  (-0.41,0.19) |
| Ethiopia | 14.08  (11.28,17.21) | 1.27 | 6.17  (5.09,7.73) | 1.14 | -0.56  (-0.65,-0.44) |  | 14.65  (11.81,18.03) | 1.30 | 6.64  (5.45,8.34) | 1.16 | -0.55  (-0.64,-0.42) |  | 398.08  (317.51,483.58) | 1.18 | 155.38  (126.23,196.84) | 1.09 | -0.61  (-0.69,-0.49) |
| Fiji | 8.36  (6.89,10.19) | 1.34 | 7.02  (5.63,8.76) | 1.52 | -0.16  (-0.36,0.13) |  | 8.95  (7.43,10.88) | 1.31 | 7.44  (5.99,9.19) | 1.50 | -0.17  (-0.36,0.10) |  | 209.14  (172.03,257.35) | 1.37 | 168.14  (132.04,213.77) | 1.54 | -0.20  (-0.40,0.10) |
| Finland | 16.29  (15.52,17.02) | 1.74 | 7.17  (5.60,8.98) | 1.63 | -0.56  (-0.66,-0.44) |  | 13.35  (12.66,13.89) | 1.78 | 4.78  (4.35,5.20) | 1.68 | -0.64  (-0.67,-0.61) |  | 289.30  (277.50,299.89) | 1.76 | 100.02  (92.07,109.13) | 1.65 | -0.65  (-0.68,-0.62) |
| France | 11.07  (10.48,11.56) | 2.46 | 6.93  (5.38,8.78) | 2.48 | -0.37  (-0.51,-0.21) |  | 10.08  (9.50,10.50) | 2.36 | 4.86  (4.36,5.27) | 2.31 | -0.52  (-0.55,-0.48) |  | 205.41  (197.41,212.92) | 2.53 | 100.94  (92.69,108.82) | 2.46 | -0.51  (-0.54,-0.47) |
| Gabon | 12.47  (10.19,15.43) | 2.17 | 7.68  (6.02,9.42) | 2.42 | -0.38  (-0.54,-0.21) |  | 13.22  (10.80,16.38) | 2.17 | 8.15  (6.43,9.99) | 2.38 | -0.38  (-0.53,-0.21) |  | 325.87  (263.30,403.96) | 2.20 | 188.35  (143.70,234.29) | 2.50 | -0.42  (-0.58,-0.24) |
| Gambia | 6.02  (4.78,7.49) | 1.98 | 5.21  (4.14,6.41) | 1.85 | -0.13  (-0.37,0.17) |  | 6.68  (5.34,8.26) | 1.98 | 5.81  (4.64,7.07) | 1.86 | -0.13  (-0.36,0.15) |  | 138.90  (107.73,178.19) | 1.98 | 118.01  (92.02,148.17) | 1.84 | -0.15  (-0.40,0.20) |
| Georgia | 19.93  (18.27,21.74) | 2.05 | 14.37  (12.09,17.01) | 2.32 | -0.28  (-0.40,-0.14) |  | 19.46  (17.82,21.19) | 2.03 | 14.07  (11.86,16.62) | 2.31 | -0.28  (-0.40,-0.14) |  | 522.92  (478.14,572.40) | 2.16 | 355.53  (298.08,423.78) | 2.41 | -0.32  (-0.44,-0.18) |
| Germany | 16.37  (15.57,17.00) | 1.94 | 9.99  (7.75,12.87) | 1.74 | -0.39  (-0.53,-0.21) |  | 13.13  (12.38,13.61) | 1.90 | 6.86  (6.29,7.40) | 1.77 | -0.48  (-0.51,-0.44) |  | 280.39  (269.93,289.02) | 1.89 | 142.04  (131.98,152.64) | 1.80 | -0.49  (-0.53,-0.45) |
| Ghana | 11.19  (9.10,13.48) | 1.14 | 7.82  (6.24,9.74) | 0.90 | -0.30  (-0.45,-0.10) |  | 12.28  (10.02,14.73) | 1.18 | 8.65  (6.92,10.76) | 0.90 | -0.30  (-0.45,-0.10) |  | 267.99  (214.32,326.70) | 1.06 | 175.57  (137.71,221.09) | 0.90 | -0.34  (-0.50,-0.14) |
| Greece | 17.00  (16.10,17.84) | 1.70 | 11.03  (8.67,13.97) | 1.93 | -0.35  (-0.49,-0.18) |  | 14.48  (13.76,15.10) | 1.63 | 8.05  (7.39,8.65) | 1.83 | -0.44  (-0.48,-0.40) |  | 304.81  (293.29,316.20) | 1.76 | 169.84  (157.88,181.57) | 1.92 | -0.44  (-0.48,-0.40) |
| Greenland | 18.83  (16.57,21.47) | 2.19 | 11.26  (9.25,13.40) | 2.48 | -0.40  (-0.52,-0.27) |  | 19.00  (16.86,21.50) | 2.22 | 10.82  (8.84,12.93) | 2.44 | -0.43  (-0.55,-0.30) |  | 469.36  (406.32,542.94) | 2.11 | 251.43  (202.06,306.03) | 2.40 | -0.46  (-0.59,-0.33) |
| Grenada | 15.73  (14.15,17.26) | 1.81 | 10.36  (9.31,11.53) | 1.38 | -0.34  (-0.43,-0.25) |  | 16.43  (14.79,18.00) | 1.80 | 10.43  (9.38,11.58) | 1.35 | -0.37  (-0.45,-0.28) |  | 379.90  (340.98,417.04) | 1.79 | 225.92  (201.14,253.23) | 1.39 | -0.41  (-0.49,-0.32) |
| Guam | 8.50  (7.50,9.54) | 1.58 | 6.09  (5.08,7.26) | 1.89 | -0.28  (-0.42,-0.12) |  | 8.91  (7.84,9.99) | 1.44 | 5.74  (4.82,6.79) | 1.76 | -0.36  (-0.47,-0.21) |  | 185.37  (163.01,208.43) | 1.73 | 144.10  (119.42,172.12) | 2.02 | -0.22  (-0.38,-0.03) |
| Guatemala | 29.72  (26.66,33.26) | 0.81 | 27.21  (21.64,33.73) | 1.00 | -0.08  (-0.28,0.16) |  | 32.28  (28.98,35.34) | 0.80 | 27.97  (22.45,34.43) | 1.03 | -0.13  (-0.31,0.08) |  | 716.58  (638.98,792.69) | 0.83 | 615.86  (483.59,770.73) | 1.02 | -0.14  (-0.33,0.10) |
| Guinea | 15.42  (13.08,17.99) | 1.36 | 15.00  (11.88,18.58) | 1.75 | -0.03  (-0.25,0.24) |  | 16.77  (14.34,19.39) | 1.39 | 16.24  (12.95,20.00) | 1.77 | -0.03  (-0.25,0.22) |  | 385.36  (325.59,453.61) | 1.29 | 370.50  (289.50,465.66) | 1.70 | -0.04  (-0.27,0.25) |
| Guinea-Bissau | 24.76  (19.57,30.13) | 2.23 | 18.04  (14.30,22.22) | 1.77 | -0.27  (-0.45,-0.06) |  | 26.07  (20.65,31.56) | 2.25 | 19.24  (15.38,23.58) | 1.78 | -0.26  (-0.44,-0.05) |  | 632.38  (493.86,786.32) | 2.22 | 436.33  (339.66,544.82) | 1.80 | -0.31  (-0.49,-0.08) |
| Guyana | 14.22  (12.46,16.06) | 1.57 | 8.60  (6.81,10.71) | 1.50 | -0.40  (-0.53,-0.22) |  | 15.00  (13.19,16.93) | 1.54 | 8.87  (7.07,11.02) | 1.47 | -0.41  (-0.54,-0.25) |  | 352.60  (306.99,399.77) | 1.60 | 213.41  (165.97,268.79) | 1.53 | -0.39  (-0.54,-0.21) |
| Haiti | 22.52  (15.79,26.65) | 1.50 | 14.99  (9.68,20.05) | 1.36 | -0.33  (-0.50,-0.11) |  | 23.95  (17.20,28.59) | 1.51 | 15.99  (10.48,21.19) | 1.37 | -0.33  (-0.49,-0.11) |  | 587.82  (401.73,703.55) | 1.42 | 374.47  (237.70,505.82) | 1.32 | -0.36  (-0.53,-0.13) |
| Honduras | 13.01  (10.76,15.11) | 1.21 | 15.47  (12.69,19.60) | 1.17 | 0.19  (-0.05,0.51) |  | 13.56  (11.17,15.78) | 1.25 | 15.88  (13.16,20.02) | 1.21 | 0.17  (-0.05,0.49) |  | 342.52  (283.21,401.73) | 1.17 | 350.34  (276.43,455.70) | 1.14 | 0.02  (-0.20,0.33) |
| Hungary | 19.94  (19.17,20.70) | 2.24 | 9.60  (7.95,11.49) | 2.09 | -0.52  (-0.60,-0.42) |  | 20.05  (19.24,20.79) | 2.22 | 8.57  (7.11,10.20) | 2.12 | -0.57  (-0.65,-0.49) |  | 443.10  (428.21,458.79) | 2.29 | 189.49  (154.81,229.51) | 2.12 | -0.57  (-0.65,-0.48) |
| Iceland | 16.54  (15.16,17.99) | 1.99 | 7.23  (6.28,8.31) | 2.55 | -0.56  (-0.63,-0.49) |  | 12.98  (12.05,13.91) | 1.97 | 4.59  (4.05,5.12) | 2.40 | -0.65  (-0.69,-0.60) |  | 274.61  (255.58,294.74) | 1.89 | 94.18  (84.31,104.77) | 2.44 | -0.66  (-0.70,-0.61) |
| India | 10.31  (9.16,11.50) | 1.11 | 7.14  (6.12,8.27) | 1.04 | -0.31  (-0.42,-0.18) |  | 10.85  (9.63,12.08) | 1.13 | 7.32  (6.30,8.44) | 1.04 | -0.33  (-0.43,-0.21) |  | 274.65  (242.83,303.76) | 1.05 | 186.96  (159.73,217.03) | 0.99 | -0.32  (-0.43,-0.20) |
| Indonesia | 8.51  (7.00,9.76) | 1.26 | 6.40  (5.36,7.28) | 1.54 | -0.25  (-0.37,-0.12) |  | 9.03  (7.47,10.32) | 1.28 | 6.82  (5.74,7.68) | 1.54 | -0.25  (-0.37,-0.11) |  | 220.16  (179.31,251.23) | 1.20 | 147.12  (122.19,170.23) | 1.52 | -0.33  (-0.45,-0.20) |
| Iran  (Islamic Republic of) | 23.93  (20.81,26.80) | 1.81 | 16.79  (15.49,18.16) | 1.87 | -0.30  (-0.38,-0.18) |  | 25.25  (22.45,28.55) | 1.79 | 16.17  (14.86,17.40) | 1.85 | -0.36  (-0.44,-0.25) |  | 564.98  (500.44,620.99) | 1.74 | 338.69  (318.54,363.06) | 1.88 | -0.40  (-0.47,-0.29) |
| Iraq | 6.71  (5.44,8.26) | 1.76 | 5.78  (4.53,7.07) | 1.63 | -0.14  (-0.36,0.14) |  | 6.99  (5.70,8.53) | 1.82 | 5.71  (4.55,6.89) | 1.72 | -0.18  (-0.39,0.06) |  | 172.23  (137.30,214.33) | 1.58 | 137.14  (104.56,171.14) | 1.45 | -0.20  (-0.41,0.09) |
| Ireland | 14.31  (13.56,15.05) | 1.89 | 8.73  (6.80,10.94) | 1.84 | -0.39  (-0.53,-0.23) |  | 12.87  (12.17,13.48) | 1.86 | 5.79  (5.25,6.32) | 1.74 | -0.55  (-0.59,-0.51) |  | 266.08  (254.62,277.78) | 1.91 | 113.28  (103.48,122.55) | 1.77 | -0.57  (-0.61,-0.54) |
| Israel | 11.03  (10.31,11.66) | 1.74 | 7.71  (6.04,9.80) | 1.84 | -0.30  (-0.45,-0.11) |  | 10.87  (10.12,11.48) | 1.76 | 6.04  (5.48,6.52) | 1.78 | -0.44  (-0.49,-0.40) |  | 223.91  (211.33,235.36) | 1.72 | 125.16  (115.77,134.68) | 1.76 | -0.44  (-0.49,-0.40) |
| Italy | 21.85  (20.97,22.49) | 1.91 | 12.74  (10.43,15.13) | 1.85 | -0.42  (-0.52,-0.31) |  | 18.56  (17.80,19.00) | 1.87 | 8.26  (7.50,8.72) | 1.86 | -0.55  (-0.58,-0.54) |  | 386.92  (377.63,393.89) | 1.95 | 167.61  (157.39,175.22) | 1.87 | -0.57  (-0.59,-0.55) |
| Jamaica | 13.94  (13.02,14.86) | 2.13 | 9.76  (7.75,12.14) | 2.16 | -0.30  (-0.45,-0.12) |  | 14.39  (13.38,15.37) | 2.07 | 9.53  (7.62,11.78) | 2.09 | -0.34  (-0.47,-0.17) |  | 307.97  (287.13,329.29) | 2.16 | 212.49  (164.12,269.37) | 2.21 | -0.31  (-0.46,-0.12) |
| Japan | 61.24  (58.81,62.71) | 2.32 | 28.29  (23.71,33.27) | 2.50 | -0.54  (-0.61,-0.46) |  | 32.19  (30.55,33.01) | 2.25 | 14.07  (12.45,15.01) | 2.54 | -0.56  (-0.60,-0.54) |  | 726.86  (704.25,740.39) | 2.10 | 282.64  (260.78,296.59) | 2.37 | -0.61  (-0.63,-0.60) |
| Jordan | 7.90  (6.64,9.36) | 1.42 | 5.34  (4.45,6.41) | 1.37 | -0.32  (-0.48,-0.14) |  | 8.34  (7.04,9.86) | 1.43 | 5.14  (4.31,6.12) | 1.35 | -0.38  (-0.53,-0.21) |  | 190.24  (159.39,225.40) | 1.34 | 109.51  (90.99,132.12) | 1.35 | -0.42  (-0.57,-0.25) |
| Kazakhstan | 33.82  (32.20,35.39) | 2.34 | 14.63  (12.77,16.69) | 2.53 | -0.57  (-0.63,-0.50) |  | 33.94  (32.27,35.51) | 2.32 | 14.11  (12.33,16.09) | 2.58 | -0.58  (-0.64,-0.52) |  | 885.31  (842.87,926.58) | 2.39 | 345.97  (300.28,398.30) | 2.50 | -0.61  (-0.67,-0.55) |
| Kenya | 8.73  (7.05,10.40) | 1.31 | 8.65  (7.04,10.33) | 1.64 | -0.01  (-0.17,0.18) |  | 9.32  (7.47,11.07) | 1.35 | 9.83  (8.11,11.80) | 1.52 | 0.05  (-0.11,0.25) |  | 219.81  (175.15,269.22) | 1.26 | 227.83  (186.27,277.73) | 1.49 | 0.04  (-0.15,0.24) |
| Kiribati | 18.54  (15.28,22.11) | 3.20 | 15.90  (12.53,19.69) | 3.11 | -0.14  (-0.35,0.13) |  | 19.41  (16.06,22.99) | 3.11 | 16.60  (13.28,20.33) | 2.93 | -0.14  (-0.34,0.12) |  | 535.70  (432.68,649.73) | 3.42 | 440.98  (341.85,554.55) | 3.42 | -0.18  (-0.39,0.11) |
| Kuwait | 6.33  (5.64,7.09) | 1.43 | 3.94  (3.26,4.76) | 1.80 | -0.38  (-0.49,-0.24) |  | 6.27  (5.55,7.04) | 1.43 | 3.45  (2.86,4.15) | 1.78 | -0.45  (-0.54,-0.34) |  | 129.87  (117.09,144.73) | 1.36 | 64.88  (54.13,77.72) | 1.77 | -0.50  (-0.59,-0.39) |
| Kyrgyzstan | 32.03  (30.35,33.80) | 2.70 | 16.97  (14.81,19.24) | 2.93 | -0.47  (-0.54,-0.39) |  | 31.81  (30.10,33.59) | 2.68 | 16.78  (14.69,19.02) | 2.89 | -0.47  (-0.54,-0.40) |  | 900.56  (853.47,951.43) | 2.75 | 433.57  (377.02,491.90) | 3.02 | -0.52  (-0.59,-0.45) |
| Lao People's Democratic Republic | 15.96  (12.28,19.77) | 1.48 | 7.77  (5.95,9.66) | 1.47 | -0.51  (-0.63,-0.37) |  | 16.76  (12.83,20.73) | 1.52 | 8.27  (6.40,10.19) | 1.48 | -0.51  (-0.62,-0.36) |  | 438.08  (330.19,548.01) | 1.42 | 191.14  (144.15,242.00) | 1.43 | -0.56  (-0.68,-0.42) |
| Latvia | 26.72  (25.70,27.74) | 1.98 | 14.05  (11.76,16.93) | 1.81 | -0.47  (-0.56,-0.36) |  | 25.11  (24.14,26.03) | 2.15 | 11.56  (9.72,13.89) | 2.20 | -0.54  (-0.62,-0.45) |  | 652.98  (628.47,677.93) | 2.17 | 278.27  (231.43,337.91) | 2.18 | -0.57  (-0.65,-0.49) |
| Lebanon | 9.81  (8.20,11.80) | 1.41 | 8.07  (6.53,10.55) | 1.60 | -0.18  (-0.38,0.18) |  | 10.18  (8.53,12.22) | 1.42 | 6.78  (5.51,8.97) | 1.61 | -0.33  (-0.49,-0.03) |  | 233.54  (194.44,284.34) | 1.33 | 149.24  (118.92,193.63) | 1.54 | -0.36  (-0.53,-0.09) |
| Lesotho | 9.92  (7.96,12.08) | 2.38 | 11.52  (8.72,14.67) | 2.25 | 0.16  (-0.13,0.54) |  | 10.67  (8.60,12.89) | 2.34 | 12.35  (9.40,15.57) | 2.18 | 0.16  (-0.13,0.53) |  | 249.71  (199.14,307.94) | 2.51 | 300.62  (224.48,391.01) | 2.47 | 0.20  (-0.13,0.63) |
| Liberia | 15.01  (12.58,17.69) | 1.49 | 11.13  (8.52,14.25) | 1.33 | -0.26  (-0.43,-0.01) |  | 16.47  (13.89,19.36) | 1.51 | 12.33  (9.54,15.64) | 1.34 | -0.25  (-0.42,-0.01) |  | 360.74  (297.50,432.49) | 1.45 | 253.92  (190.68,333.92) | 1.32 | -0.30  (-0.47,-0.04) |
| Libya | 7.09  (5.33,8.89) | 1.50 | 5.70  (4.39,7.23) | 1.58 | -0.20  (-0.43,0.16) |  | 7.37  (5.56,9.23) | 1.54 | 5.64  (4.36,7.12) | 1.61 | -0.23  (-0.45,0.10) |  | 171.36  (129.39,216.31) | 1.39 | 128.48  (98.02,166.36) | 1.49 | -0.25  (-0.48,0.11) |
| Lithuania | 26.51  (25.52,27.50) | 2.10 | 13.93  (11.27,17.01) | 1.94 | -0.47  (-0.58,-0.36) |  | 24.57  (23.61,25.49) | 2.36 | 11.66  (9.45,14.13) | 2.39 | -0.53  (-0.62,-0.42) |  | 627.34  (603.11,651.81) | 2.36 | 281.06  (223.86,345.15) | 2.31 | -0.55  (-0.64,-0.45) |
| Luxembourg | 13.84  (12.95,14.91) | 2.14 | 6.68  (5.45,8.07) | 2.21 | -0.52  (-0.61,-0.41) |  | 12.46  (11.66,13.24) | 2.09 | 4.75  (4.07,5.42) | 2.03 | -0.62  (-0.67,-0.56) |  | 261.99  (246.23,277.85) | 2.13 | 94.06  (81.30,107.19) | 2.06 | -0.64  (-0.69,-0.59) |
| Madagascar | 8.82  (7.54,10.16) | 1.60 | 6.58  (5.03,8.46) | 1.41 | -0.25  (-0.44,-0.02) |  | 9.41  (8.08,10.86) | 1.66 | 7.02  (5.40,8.95) | 1.43 | -0.25  (-0.44,-0.03) |  | 238.62  (202.78,275.31) | 1.47 | 172.45  (128.25,223.67) | 1.32 | -0.28  (-0.47,-0.04) |
| Malawi | 4.76  (4.09,5.50) | 1.84 | 3.28  (2.67,3.91) | 2.13 | -0.31  (-0.45,-0.14) |  | 5.23  (4.48,6.02) | 1.86 | 3.60  (2.96,4.24) | 2.10 | -0.31  (-0.44,-0.15) |  | 113.69  (96.71,133.51) | 1.68 | 77.34  (61.45,94.32) | 2.09 | -0.32  (-0.47,-0.13) |
| Malaysia | 9.77  (8.96,10.66) | 1.40 | 7.24  (5.86,8.96) | 1.40 | -0.26  (-0.41,-0.08) |  | 10.27  (9.42,11.21) | 1.38 | 7.04  (5.73,8.64) | 1.35 | -0.31  (-0.45,-0.16) |  | 234.01  (214.38,255.13) | 1.38 | 146.16  (117.08,181.22) | 1.43 | -0.38  (-0.51,-0.22) |
| Maldives | 9.32  (7.29,10.86) | 0.89 | 3.79  (3.11,4.52) | 1.13 | -0.59  (-0.68,-0.46) |  | 9.95  (7.80,11.50) | 0.90 | 3.60  (2.98,4.26) | 1.08 | -0.64  (-0.71,-0.52) |  | 229.05  (176.65,270.44) | 0.85 | 68.10  (56.00,81.23) | 1.13 | -0.70  (-0.77,-0.60) |
| Mali | 21.85  (18.38,25.54) | 1.36 | 17.12  (13.74,21.49) | 1.36 | -0.22  (-0.38,0.00) |  | 23.11  (19.56,26.85) | 1.40 | 18.22  (14.75,22.65) | 1.39 | -0.21  (-0.37,0.00) |  | 580.02  (482.74,685.46) | 1.28 | 435.20  (339.55,555.19) | 1.30 | -0.25  (-0.42,-0.01) |
| Malta | 12.92  (11.77,13.95) | 2.30 | 6.79  (5.71,7.93) | 2.50 | -0.47  (-0.56,-0.37) |  | 11.94  (10.99,12.91) | 2.30 | 4.93  (4.34,5.55) | 2.41 | -0.59  (-0.64,-0.53) |  | 246.33  (228.92,264.46) | 2.23 | 100.71  (89.69,113.25) | 2.36 | -0.59  (-0.64,-0.53) |
| Marshall Islands | 19.93  (16.64,23.10) | 2.07 | 16.25  (12.50,20.54) | 1.59 | -0.18  (-0.37,0.03) |  | 21.13  (17.66,24.50) | 2.02 | 16.99  (13.14,21.22) | 1.52 | -0.20  (-0.37,0.02) |  | 527.48  (439.51,612.48) | 2.15 | 429.16  (327.41,551.87) | 1.71 | -0.19  (-0.38,0.05) |
| Mauritania | 16.57  (13.98,19.59) | 1.41 | 10.16  (7.86,12.86) | 1.12 | -0.39  (-0.52,-0.22) |  | 18.01  (15.23,21.10) | 1.42 | 11.29  (8.81,14.13) | 1.13 | -0.37  (-0.51,-0.22) |  | 405.62  (337.40,481.50) | 1.38 | 221.83  (165.40,289.21) | 1.11 | -0.45  (-0.59,-0.28) |
| Mauritius | 14.54  (13.71,15.40) | 1.90 | 7.56  (6.15,9.19) | 1.82 | -0.48  (-0.58,-0.37) |  | 14.94  (14.07,15.82) | 1.83 | 7.14  (5.86,8.62) | 1.76 | -0.52  (-0.61,-0.42) |  | 338.93  (320.45,360.31) | 1.92 | 159.00  (128.15,194.05) | 1.79 | -0.53  (-0.62,-0.42) |
| Mexico | 13.69  (13.02,14.11) | 1.15 | 9.70  (8.43,11.19) | 1.30 | -0.29  (-0.38,-0.19) |  | 14.46  (13.68,14.93) | 1.13 | 8.86  (7.64,10.16) | 1.35 | -0.39  (-0.46,-0.29) |  | 305.23  (296.01,312.37) | 1.23 | 202.96  (174.57,234.27) | 1.39 | -0.34  (-0.42,-0.22) |
| Micronesia  (Federated States of) | 19.79  (15.76,24.27) | 1.85 | 16.85  (12.61,21.13) | 1.80 | -0.15  (-0.39,0.16) |  | 20.85  (16.63,25.54) | 1.78 | 17.40  (13.30,21.65) | 1.69 | -0.17  (-0.40,0.12) |  | 538.56  (423.09,664.46) | 1.99 | 436.36  (305.18,560.42) | 2.00 | -0.19  (-0.45,0.13) |
| Monaco | 14.57  (11.56,17.52) | 2.14 | 9.91  (7.82,12.01) | 2.19 | -0.32  (-0.47,-0.11) |  | 11.44  (9.11,13.60) | 2.06 | 6.62  (5.31,7.86) | 2.06 | -0.42  (-0.55,-0.26) |  | 241.68  (192.08,293.83) | 2.14 | 135.48  (107.82,165.44) | 2.17 | -0.44  (-0.57,-0.25) |
| Mongolia | 65.41  (54.92,77.35) | 1.73 | 43.70  (34.29,55.10) | 2.34 | -0.33  (-0.49,-0.13) |  | 68.82  (58.12,80.89) | 1.76 | 46.04  (36.30,57.48) | 2.36 | -0.33  (-0.48,-0.14) |  | 1674.72  (1385.26,2004.84) | 1.66 | 1059.24  (816.79,1351.35) | 2.37 | -0.37  (-0.53,-0.16) |
| Montenegro | 9.12  (8.03,10.14) | 2.23 | 8.24  (6.88,9.78) | 2.27 | -0.10  (-0.26,0.11) |  | 8.73  (7.68,9.71) | 2.26 | 7.47  (6.28,8.82) | 2.30 | -0.14  (-0.30,0.05) |  | 204.05  (180.56,227.20) | 2.26 | 165.75  (137.94,197.40) | 2.28 | -0.19  (-0.34,0.00) |
| Morocco | 5.40  (4.09,6.17) | 1.61 | 4.60  (3.54,5.47) | 1.65 | -0.15  (-0.34,0.07) |  | 5.74  (4.29,6.54) | 1.61 | 4.84  (3.76,5.72) | 1.68 | -0.16  (-0.34,0.05) |  | 129.53  (101.70,147.85) | 1.44 | 102.67  (78.06,125.63) | 1.48 | -0.21  (-0.40,0.02) |
| Mozambique | 6.75  (5.60,7.92) | 1.76 | 6.93  (5.51,8.66) | 2.21 | 0.03  (-0.21,0.31) |  | 7.48  (6.23,8.74) | 1.78 | 7.59  (6.06,9.42) | 2.17 | 0.01  (-0.21,0.29) |  | 162.25  (131.57,192.68) | 1.77 | 166.92  (128.77,211.63) | 2.31 | 0.03  (-0.23,0.36) |
| Myanmar | 14.17  (11.08,18.22) | 1.55 | 6.86  (5.75,8.30) | 1.73 | -0.52  (-0.64,-0.38) |  | 14.93  (11.79,18.68) | 1.58 | 7.22  (6.10,8.66) | 1.72 | -0.52  (-0.63,-0.37) |  | 388.29  (302.80,487.46) | 1.45 | 166.68  (135.60,206.26) | 1.71 | -0.57  (-0.68,-0.42) |
| Namibia | 4.12  (3.32,4.83) | 1.76 | 3.48  (2.83,4.28) | 1.93 | -0.16  (-0.34,0.10) |  | 4.53  (3.67,5.26) | 1.79 | 3.80  (3.11,4.63) | 1.90 | -0.16  (-0.34,0.08) |  | 100.38  (79.23,120.08) | 1.70 | 79.73  (62.38,100.84) | 2.03 | -0.21  (-0.40,0.09) |
| Nauru | 20.35  (16.26,25.04) | 1.89 | 17.90  (14.57,21.72) | 2.05 | -0.12  (-0.29,0.09) |  | 21.03  (16.96,25.84) | 1.79 | 17.94  (14.73,21.60) | 1.92 | -0.15  (-0.31,0.05) |  | 535.58  (422.22,670.49) | 2.06 | 451.74  (359.71,557.16) | 2.25 | -0.16  (-0.33,0.07) |
| Nepal | 9.57  (7.40,11.86) | 1.19 | 8.42  (6.58,10.66) | 1.13 | -0.12  (-0.34,0.19) |  | 10.10  (7.96,12.46) | 1.24 | 8.96  (7.05,11.34) | 1.15 | -0.11  (-0.34,0.19) |  | 262.76  (204.90,327.65) | 1.10 | 208.61  (160.81,267.15) | 1.06 | -0.21  (-0.42,0.09) |
| Netherlands | 15.11  (14.33,15.80) | 2.35 | 10.23  (8.04,12.75) | 1.78 | -0.32  (-0.47,-0.15) |  | 12.47  (11.80,13.00) | 2.25 | 6.86  (6.20,7.45) | 1.70 | -0.45  (-0.49,-0.41) |  | 257.10  (247.25,266.39) | 2.29 | 131.48  (121.19,141.77) | 1.70 | -0.49  (-0.53,-0.45) |
| New Zealand | 10.97  (10.30,11.61) | 1.76 | 6.96  (5.75,8.31) | 1.72 | -0.37  (-0.47,-0.24) |  | 9.02  (8.51,9.52) | 1.92 | 4.65  (4.26,4.98) | 1.87 | -0.48  (-0.52,-0.44) |  | 200.40  (190.56,210.73) | 1.82 | 101.24  (94.74,107.59) | 1.79 | -0.49  (-0.53,-0.45) |
| Nicaragua | 15.75  (14.23,17.25) | 1.95 | 15.77  (12.99,18.74) | 1.88 | 0.00  (-0.18,0.20) |  | 16.41  (14.84,17.95) | 2.10 | 15.03  (12.54,17.62) | 1.95 | -0.08  (-0.24,0.08) |  | 384.84  (351.64,420.02) | 1.81 | 316.51  (256.78,381.73) | 2.00 | -0.18  (-0.34,0.00) |
| Niger | 15.95  (12.99,18.58) | 1.57 | 13.26  (10.13,16.66) | 1.36 | -0.17  (-0.33,0.07) |  | 17.36  (14.09,20.19) | 1.59 | 14.68  (11.25,18.24) | 1.37 | -0.15  (-0.32,0.08) |  | 392.70  (317.87,463.28) | 1.54 | 307.09  (231.92,391.48) | 1.36 | -0.22  (-0.39,0.03) |
| Nigeria | 4.55  (3.61,5.65) | 1.48 | 3.91  (3.20,4.77) | 1.40 | -0.14  (-0.34,0.11) |  | 5.05  (4.01,6.22) | 1.49 | 4.43  (3.60,5.42) | 1.40 | -0.12  (-0.31,0.14) |  | 105.82  (83.08,133.85) | 1.47 | 85.70  (67.57,108.09) | 1.41 | -0.19  (-0.38,0.08) |
| Niue | 13.35  (11.07,16.12) | 2.08 | 11.01  (9.04,13.30) | 2.04 | -0.18  (-0.36,0.06) |  | 13.74  (11.47,16.49) | 1.97 | 10.43  (8.58,12.41) | 1.92 | -0.24  (-0.40,-0.04) |  | 331.47  (266.79,406.59) | 2.24 | 241.27  (191.03,303.26) | 2.16 | -0.27  (-0.46,-0.02) |
| North Macedonia | 22.53  (20.75,24.02) | 1.98 | 16.15  (12.88,20.23) | 2.14 | -0.28  (-0.44,-0.10) |  | 22.81  (20.98,24.35) | 1.97 | 15.32  (12.30,19.13) | 2.16 | -0.33  (-0.46,-0.16) |  | 555.04  (511.73,593.62) | 1.99 | 345.84  (271.39,434.50) | 2.16 | -0.38  (-0.51,-0.21) |
| Northern Mariana Islands | 18.78  (15.84,22.47) | 1.06 | 13.01  (11.03,15.13) | 1.51 | -0.31  (-0.45,-0.15) |  | 18.79  (15.89,22.34) | 0.99 | 12.02  (10.31,13.89) | 1.40 | -0.36  (-0.49,-0.22) |  | 432.97  (355.93,526.99) | 1.10 | 270.49  (224.41,322.76) | 1.60 | -0.38  (-0.52,-0.22) |
| Norway | 12.96  (12.36,13.50) | 2.00 | 6.67  (5.65,7.82) | 1.70 | -0.49  (-0.56,-0.40) |  | 10.73  (10.20,11.08) | 1.99 | 4.49  (4.12,4.82) | 1.64 | -0.58  (-0.61,-0.55) |  | 227.61  (220.29,233.86) | 1.92 | 89.00  (82.72,95.46) | 1.61 | -0.61  (-0.63,-0.58) |
| Oman | 12.88  (9.75,16.13) | 1.61 | 8.55  (7.51,9.82) | 1.52 | -0.34  (-0.49,-0.09) |  | 13.31  (10.19,16.57) | 1.63 | 7.98  (6.95,9.13) | 1.56 | -0.40  (-0.53,-0.18) |  | 301.72  (224.83,385.17) | 1.53 | 152.34  (132.54,175.90) | 1.41 | -0.50  (-0.62,-0.30) |
| Pakistan | 6.58  (5.23,7.74) | 1.83 | 6.45  (5.34,7.79) | 1.78 | -0.02  (-0.21,0.28) |  | 7.13  (5.66,8.46) | 1.87 | 6.87  (5.71,8.31) | 1.80 | -0.04  (-0.22,0.24) |  | 165.67  (134.80,193.87) | 1.73 | 162.70  (133.94,198.78) | 1.70 | -0.02  (-0.22,0.28) |
| Palau | 13.92  (10.99,17.43) | 4.45 | 12.25  (9.69,15.32) | 4.66 | -0.12  (-0.35,0.18) |  | 13.74  (10.91,17.10) | 4.13 | 11.10  (8.87,13.73) | 4.22 | -0.19  (-0.39,0.08) |  | 357.23  (274.78,456.27) | 4.92 | 293.28  (227.35,376.79) | 5.21 | -0.18  (-0.41,0.14) |
| Palestine | 11.04  (8.53,14.14) | 1.72 | 7.16  (6.13,8.27) | 1.52 | -0.35  (-0.51,-0.12) |  | 11.47  (8.91,14.52) | 1.76 | 7.12  (6.13,8.22) | 1.57 | -0.38  (-0.53,-0.17) |  | 272.02  (207.72,351.29) | 1.59 | 160.58  (136.75,187.31) | 1.43 | -0.41  (-0.56,-0.19) |
| Panama | 17.64  (16.44,18.70) | 1.84 | 12.47  (9.70,15.94) | 1.63 | -0.29  (-0.45,-0.10) |  | 17.26  (16.04,18.31) | 1.92 | 10.76  (8.45,13.63) | 1.80 | -0.38  (-0.51,-0.21) |  | 409.96  (383.43,432.67) | 1.85 | 243.97  (187.57,314.14) | 1.65 | -0.40  (-0.54,-0.23) |
| Papua New Guinea | 13.59  (10.17,17.47) | 2.05 | 13.26  (9.68,17.25) | 1.83 | -0.02  (-0.24,0.26) |  | 14.34  (10.82,18.27) | 2.00 | 13.95  (10.27,18.04) | 1.77 | -0.03  (-0.23,0.23) |  | 355.66  (263.07,462.94) | 2.08 | 343.02  (244.56,453.02) | 1.92 | -0.04  (-0.27,0.27) |
| Paraguay | 10.26  (9.06,11.52) | 1.73 | 8.91  (6.85,11.42) | 2.22 | -0.13  (-0.34,0.15) |  | 10.78  (9.51,12.06) | 1.71 | 8.72  (6.76,11.08) | 2.19 | -0.19  (-0.38,0.06) |  | 246.28  (217.29,277.69) | 1.72 | 199.48  (151.26,257.39) | 2.25 | -0.19  (-0.40,0.09) |
| Peru | 25.38  (21.96,29.26) | 1.43 | 19.56  (14.67,25.59) | 1.40 | -0.23  (-0.44,0.05) |  | 26.81  (23.21,30.85) | 1.38 | 17.81  (13.44,23.13) | 1.20 | -0.34  (-0.51,-0.10) |  | 613.97  (532.34,712.02) | 1.43 | 388.08  (288.38,515.23) | 1.26 | -0.37  (-0.55,-0.13) |
| Philippines | 7.05  (6.27,7.92) | 1.62 | 4.38  (3.62,5.27) | 1.80 | -0.38  (-0.51,-0.21) |  | 7.46  (6.66,8.30) | 1.54 | 4.50  (3.78,5.30) | 1.74 | -0.40  (-0.51,-0.26) |  | 178.50  (159.42,199.66) | 1.73 | 107.44  (88.82,128.45) | 1.84 | -0.40  (-0.52,-0.26) |
| Poland | 18.85  (18.26,19.32) | 2.63 | 9.33  (7.89,11.08) | 2.66 | -0.51  (-0.58,-0.42) |  | 20.18  (19.42,20.71) | 2.58 | 9.66  (8.10,11.35) | 2.68 | -0.52  (-0.60,-0.44) |  | 467.60  (455.99,477.90) | 2.73 | 218.89  (181.85,260.04) | 2.73 | -0.53  (-0.61,-0.44) |
| Portugal | 28.19  (26.99,29.28) | 1.83 | 14.64  (11.41,18.59) | 1.93 | -0.48  (-0.59,-0.34) |  | 28.36  (27.09,29.46) | 1.83 | 11.95  (10.94,12.82) | 2.05 | -0.58  (-0.61,-0.55) |  | 623.06  (602.77,644.04) | 1.96 | 262.45  (242.92,281.09) | 2.12 | -0.58  (-0.61,-0.55) |
| Puerto Rico | 11.65  (10.92,12.30) | 2.26 | 5.83  (4.55,7.41) | 1.86 | -0.50  (-0.61,-0.36) |  | 11.59  (10.80,12.26) | 2.22 | 4.74  (3.70,5.97) | 1.82 | -0.59  (-0.68,-0.48) |  | 235.87  (223.14,248.19) | 2.27 | 99.21  (76.70,128.41) | 1.83 | -0.58  (-0.68,-0.45) |
| Qatar | 11.93  (9.63,14.84) | 1.25 | 8.99  (6.95,11.39) | 0.78 | -0.25  (-0.46,0.03) |  | 12.66  (10.26,15.85) | 1.28 | 8.26  (6.47,10.36) | 0.77 | -0.35  (-0.52,-0.12) |  | 258.09  (207.29,316.18) | 1.10 | 137.31  (107.04,176.98) | 0.75 | -0.47  (-0.62,-0.27) |
| Republic of Korea | 61.57  (58.72,64.45) | 2.38 | 28.67  (23.65,34.17) | 2.33 | -0.53  (-0.61,-0.44) |  | 52.35  (49.97,55.05) | 2.36 | 14.09  (12.57,15.60) | 2.37 | -0.73  (-0.76,-0.70) |  | 1360.58  (1300.37,1416.56) | 2.26 | 298.70  (270.17,328.42) | 2.23 | -0.78  (-0.80,-0.75) |
| Republic of Moldova | 20.24  (19.43,21.00) | 2.22 | 10.40  (8.97,11.94) | 2.28 | -0.49  (-0.56,-0.41) |  | 19.49  (18.71,20.23) | 2.32 | 9.32  (8.04,10.66) | 2.53 | -0.52  (-0.59,-0.45) |  | 544.65  (523.00,565.59) | 2.31 | 248.76  (213.70,287.99) | 2.56 | -0.54  (-0.61,-0.47) |
| Romania | 14.85  (14.29,15.44) | 2.51 | 11.74  (9.53,14.35) | 2.94 | -0.21  (-0.36,-0.03) |  | 14.76  (14.18,15.34) | 2.47 | 10.46  (8.56,12.70) | 2.82 | -0.29  (-0.42,-0.13) |  | 375.51  (361.29,390.62) | 2.57 | 254.63  (207.40,313.11) | 2.91 | -0.32  (-0.45,-0.17) |
| Russian Federation | 31.30  (30.03,31.95) | 2.31 | 16.08  (14.09,18.39) | 2.16 | -0.49  (-0.55,-0.42) |  | 29.48  (28.36,30.07) | 2.31 | 13.22  (11.53,15.08) | 2.19 | -0.55  (-0.60,-0.49) |  | 753.55  (719.22,770.38) | 2.36 | 320.51  (278.92,368.66) | 2.17 | -0.57  (-0.63,-0.51) |
| Rwanda | 13.15  (10.87,15.57) | 1.92 | 6.87  (5.41,8.46) | 1.77 | -0.48  (-0.59,-0.33) |  | 13.86  (11.46,16.38) | 1.97 | 7.36  (5.87,9.03) | 1.79 | -0.47  (-0.58,-0.32) |  | 359.95  (293.91,427.72) | 1.80 | 172.36  (132.50,216.56) | 1.73 | -0.52  (-0.64,-0.37) |
| Saint Kitts and Nevis | 18.60  (16.90,20.18) | 1.85 | 10.69  (8.99,12.49) | 2.21 | -0.43  (-0.52,-0.31) |  | 18.99  (17.29,20.59) | 1.83 | 9.99  (8.52,11.56) | 2.07 | -0.47  (-0.56,-0.38) |  | 431.71  (394.76,471.90) | 1.86 | 215.24  (172.58,256.31) | 2.29 | -0.50  (-0.60,-0.39) |
| Saint Lucia | 20.18  (18.70,21.65) | 2.19 | 12.17  (10.26,14.21) | 2.32 | -0.40  (-0.50,-0.28) |  | 21.28  (19.68,22.83) | 2.18 | 12.05  (10.18,14.05) | 2.28 | -0.43  (-0.52,-0.33) |  | 475.31  (440.44,510.25) | 2.15 | 266.49  (224.32,315.80) | 2.38 | -0.44  (-0.54,-0.33) |
| Saint Vincent and the Grenadines | 16.05  (14.61,17.53) | 1.79 | 11.18  (9.75,12.91) | 1.72 | -0.30  (-0.41,-0.19) |  | 16.85  (15.35,18.36) | 1.76 | 11.41  (10.02,13.14) | 1.68 | -0.32  (-0.42,-0.21) |  | 377.83  (345.06,415.02) | 1.82 | 254.75  (221.33,294.85) | 1.77 | -0.33  (-0.43,-0.21) |
| Samoa | 15.43  (12.57,18.83) | 2.01 | 12.22  (9.78,15.23) | 1.58 | -0.21  (-0.40,0.03) |  | 16.11  (13.29,19.45) | 2.01 | 12.40  (10.02,15.31) | 1.59 | -0.23  (-0.41,-0.01) |  | 387.84  (308.46,482.00) | 1.92 | 293.61  (228.46,373.82) | 1.48 | -0.24  (-0.45,0.01) |
| San Marino | 34.94  (30.04,39.86) | 2.18 | 26.16  (19.98,33.99) | 2.32 | -0.25  (-0.44,0.01) |  | 28.63  (24.22,32.90) | 2.08 | 18.55  (12.51,25.86) | 2.07 | -0.35  (-0.57,-0.06) |  | 559.88  (471.48,651.76) | 2.20 | 358.53  (233.43,518.49) | 2.08 | -0.36  (-0.60,-0.02) |
| Sao Tome and Principe | 16.50  (13.79,19.25) | 1.55 | 16.58  (13.49,20.66) | 1.66 | 0.00  (-0.20,0.31) |  | 18.31  (15.43,21.11) | 1.57 | 18.23  (14.88,22.59) | 1.64 | 0.00  (-0.21,0.29) |  | 379.48  (306.14,451.52) | 1.56 | 368.22  (296.27,463.42) | 1.72 | -0.03  (-0.26,0.31) |
| Saudi Arabia | 6.39  (4.91,7.87) | 1.66 | 4.40  (3.60,5.33) | 1.44 | -0.31  (-0.49,-0.02) |  | 7.61  (5.74,9.62) | 1.54 | 4.10  (3.36,4.95) | 1.43 | -0.46  (-0.61,-0.22) |  | 162.62  (119.18,210.61) | 1.49 | 86.23  (69.30,106.80) | 1.43 | -0.47  (-0.63,-0.21) |
| Senegal | 15.80  (12.93,18.39) | 1.85 | 12.66  (9.98,15.52) | 1.60 | -0.20  (-0.38,0.03) |  | 17.34  (14.32,20.14) | 1.87 | 14.01  (11.18,17.10) | 1.60 | -0.19  (-0.36,0.03) |  | 381.82  (307.21,449.41) | 1.84 | 288.68  (225.79,357.53) | 1.63 | -0.24  (-0.42,0.00) |
| Serbia | 13.65  (11.63,15.08) | 1.94 | 9.69  (7.71,12.09) | 2.09 | -0.29  (-0.45,-0.09) |  | 13.71  (11.66,15.15) | 1.91 | 8.70  (6.95,10.83) | 2.06 | -0.37  (-0.50,-0.19) |  | 335.18  (289.51,370.20) | 1.99 | 199.96  (155.71,251.56) | 2.09 | -0.40  (-0.54,-0.23) |
| Seychelles | 11.25  (10.02,12.77) | 2.75 | 7.15  (6.16,8.21) | 2.41 | -0.36  (-0.47,-0.25) |  | 11.54  (10.34,13.11) | 2.72 | 6.85  (5.96,7.82) | 2.33 | -0.41  (-0.50,-0.30) |  | 287.09  (254.95,327.41) | 2.64 | 161.17  (138.88,185.81) | 2.39 | -0.44  (-0.54,-0.33) |
| Sierra Leone | 14.89  (12.05,17.77) | 1.86 | 12.44  (9.67,15.78) | 1.35 | -0.16  (-0.35,0.08) |  | 16.37  (13.40,19.51) | 1.86 | 13.68  (10.73,17.25) | 1.36 | -0.16  (-0.35,0.08) |  | 357.76  (284.92,435.67) | 1.87 | 289.86  (220.92,375.78) | 1.35 | -0.19  (-0.39,0.08) |
| Singapore | 26.88  (25.30,28.36) | 2.09 | 10.72  (8.60,13.31) | 1.49 | -0.60  (-0.68,-0.50) |  | 20.41  (19.30,21.36) | 2.14 | 5.54  (4.87,6.05) | 1.56 | -0.73  (-0.75,-0.70) |  | 443.38  (422.91,462.88) | 2.03 | 105.97  (96.24,114.86) | 1.43 | -0.76  (-0.78,-0.74) |
| Slovakia | 21.12  (20.05,22.18) | 2.30 | 12.19  (9.58,15.17) | 1.99 | -0.42  (-0.55,-0.28) |  | 17.88  (16.96,18.72) | 2.34 | 8.32  (6.61,10.31) | 2.12 | -0.53  (-0.63,-0.42) |  | 421.78  (400.59,443.02) | 2.40 | 183.73  (141.73,229.94) | 2.06 | -0.56  (-0.67,-0.45) |
| Slovenia | 21.94  (17.04,27.98) | 2.10 | 11.94  (9.34,15.62) | 2.29 | -0.46  (-0.62,-0.24) |  | 20.15  (15.84,25.48) | 2.08 | 8.54  (6.71,10.95) | 2.40 | -0.58  (-0.70,-0.41) |  | 453.14  (347.74,585.85) | 2.14 | 179.29  (137.72,233.99) | 2.30 | -0.60  (-0.72,-0.44) |
| Solomon Islands | 25.10  (18.13,31.60) | 1.86 | 23.66  (18.58,28.89) | 1.75 | -0.06  (-0.28,0.24) |  | 25.64  (18.81,32.21) | 1.81 | 23.87  (18.94,29.03) | 1.67 | -0.07  (-0.28,0.22) |  | 725.55  (504.57,924.20) | 1.90 | 665.01  (494.13,827.24) | 1.85 | -0.08  (-0.32,0.22) |
| Somalia | 13.03  (9.81,16.44) | 2.17 | 9.93  (7.35,13.21) | 1.97 | -0.24  (-0.42,0.02) |  | 13.77  (10.42,17.32) | 2.21 | 10.62  (7.89,13.96) | 1.96 | -0.23  (-0.41,0.02) |  | 357.09  (263.67,457.64) | 2.06 | 273.61  (198.32,367.38) | 1.90 | -0.23  (-0.43,0.04) |
| South Africa | 8.05  (7.22,8.90) | 1.97 | 5.39  (5.00,5.83) | 2.14 | -0.33  (-0.39,-0.26) |  | 8.47  (7.57,9.34) | 1.90 | 5.70  (5.32,6.09) | 2.06 | -0.33  (-0.40,-0.25) |  | 208.57  (188.69,228.25) | 2.03 | 127.30  (118.48,137.34) | 2.28 | -0.39  (-0.45,-0.31) |
| South Sudan | 9.44  (7.04,12.27) | 1.66 | 6.72  (4.92,9.10) | 1.52 | -0.29  (-0.46,-0.05) |  | 10.18  (7.66,13.14) | 1.70 | 7.30  (5.31,9.89) | 1.54 | -0.28  (-0.46,-0.05) |  | 244.63  (178.15,323.92) | 1.59 | 169.51  (118.57,239.09) | 1.47 | -0.31  (-0.50,-0.04) |
| Spain | 19.01  (17.93,19.85) | 2.13 | 11.46  (8.95,14.37) | 2.08 | -0.40  (-0.53,-0.24) |  | 15.63  (14.74,16.28) | 1.97 | 7.02  (6.39,7.61) | 1.95 | -0.55  (-0.58,-0.52) |  | 335.78  (321.97,347.41) | 2.11 | 146.82  (136.43,158.10) | 2.00 | -0.56  (-0.59,-0.53) |
| Sri Lanka | 8.98  (8.10,10.00) | 1.15 | 5.43  (4.12,7.13) | 1.40 | -0.40  (-0.55,-0.19) |  | 9.62  (8.66,10.71) | 1.13 | 5.04  (3.87,6.55) | 1.33 | -0.48  (-0.61,-0.31) |  | 207.67  (186.82,230.64) | 1.15 | 107.38  (80.95,141.55) | 1.43 | -0.48  (-0.63,-0.30) |
| Sudan | 16.70  (11.36,20.43) | 1.66 | 14.95  (10.27,19.41) | 1.62 | -0.10  (-0.31,0.17) |  | 17.74  (12.10,21.70) | 1.70 | 15.63  (10.93,20.20) | 1.66 | -0.12  (-0.31,0.14) |  | 428.85  (286.24,527.62) | 1.53 | 354.00  (238.36,476.24) | 1.53 | -0.17  (-0.38,0.10) |
| Suriname | 9.79  (8.80,10.61) | 1.77 | 7.12  (5.94,8.54) | 1.83 | -0.27  (-0.40,-0.11) |  | 10.37  (9.29,11.25) | 1.76 | 7.28  (6.09,8.70) | 1.80 | -0.30  (-0.42,-0.15) |  | 239.98  (212.03,261.31) | 1.78 | 168.23  (138.76,202.58) | 1.84 | -0.30  (-0.43,-0.13) |
| Sweden | 11.19  (10.58,11.68) | 1.93 | 5.01  (4.20,5.86) | 1.73 | -0.55  (-0.62,-0.48) |  | 9.64  (9.06,10.06) | 1.88 | 3.67  (3.34,3.93) | 1.60 | -0.62  (-0.64,-0.59) |  | 198.74  (190.31,206.01) | 1.79 | 73.50  (68.47,78.07) | 1.56 | -0.63  (-0.65,-0.61) |
| Switzerland | 14.36  (13.59,15.14) | 2.16 | 6.64  (5.18,8.42) | 2.01 | -0.54  (-0.64,-0.41) |  | 10.77  (10.23,11.22) | 2.08 | 4.03  (3.63,4.38) | 1.88 | -0.63  (-0.66,-0.60) |  | 231.56  (222.43,240.82) | 2.12 | 83.37  (76.34,89.92) | 1.92 | -0.64  (-0.67,-0.61) |
| Syrian Arab Republic | 5.77  (4.60,7.09) | 1.38 | 4.97  (3.78,6.55) | 1.27 | -0.14  (-0.40,0.26) |  | 6.09  (4.86,7.43) | 1.36 | 4.97  (3.82,6.47) | 1.22 | -0.18  (-0.42,0.18) |  | 144.39  (113.24,178.43) | 1.36 | 107.16  (79.27,143.58) | 1.33 | -0.26  (-0.49,0.09) |
| Taiwan  (Province of China) | 19.63  (18.85,20.32) | 2.26 | 15.60  (12.23,20.26) | 2.34 | -0.21  (-0.37,0.02) |  | 17.52  (16.78,18.15) | 2.20 | 10.72  (8.42,13.73) | 2.18 | -0.39  (-0.52,-0.22) |  | 414.30  (400.35,428.31) | 2.07 | 227.64  (177.03,296.58) | 2.23 | -0.45  (-0.57,-0.29) |
| Tajikistan | 31.34  (29.05,33.97) | 2.29 | 24.01  (19.72,29.11) | 1.71 | -0.23  (-0.38,-0.06) |  | 31.67  (29.35,34.32) | 2.29 | 25.26  (20.77,30.44) | 1.73 | -0.20  (-0.35,-0.03) |  | 862.02  (796.48,940.41) | 2.25 | 590.16  (479.92,724.40) | 1.68 | -0.32  (-0.45,-0.15) |
| Thailand | 10.83  (9.54,12.17) | 1.91 | 5.90  (4.44,7.81) | 1.83 | -0.46  (-0.60,-0.26) |  | 11.11  (9.81,12.47) | 1.88 | 5.22  (3.93,6.85) | 1.77 | -0.53  (-0.65,-0.37) |  | 277.65  (243.95,313.22) | 1.89 | 129.50  (96.00,171.32) | 1.76 | -0.53  (-0.66,-0.37) |
| Timor-Leste | 10.03  (7.54,12.55) | 1.00 | 7.30  (5.36,8.99) | 1.28 | -0.27  (-0.45,-0.06) |  | 10.69  (8.04,13.29) | 1.02 | 7.85  (5.83,9.60) | 1.30 | -0.27  (-0.44,-0.05) |  | 259.92  (191.13,331.33) | 0.96 | 172.77  (123.01,217.96) | 1.25 | -0.34  (-0.51,-0.11) |
| Togo | 15.88  (13.36,18.75) | 1.66 | 13.02  (10.51,16.39) | 1.89 | -0.18  (-0.35,0.05) |  | 17.18  (14.57,20.20) | 1.66 | 14.17  (11.52,17.72) | 1.86 | -0.18  (-0.34,0.05) |  | 383.22  (316.69,460.90) | 1.63 | 303.97  (237.70,392.83) | 1.93 | -0.21  (-0.39,0.04) |
| Tokelau | 13.13  (10.55,16.28) | 1.09 | 9.68  (7.70,12.16) | 1.04 | -0.26  (-0.44,-0.04) |  | 13.94  (11.33,17.26) | 1.07 | 9.86  (8.00,12.28) | 0.99 | -0.29  (-0.46,-0.08) |  | 331.93  (261.64,425.56) | 1.08 | 226.72  (172.82,290.81) | 1.05 | -0.32  (-0.51,-0.08) |
| Tonga | 15.46  (12.74,18.25) | 2.21 | 13.34  (10.91,16.05) | 2.40 | -0.14  (-0.34,0.13) |  | 16.48  (13.63,19.40) | 2.19 | 13.85  (11.50,16.52) | 2.34 | -0.16  (-0.34,0.10) |  | 377.99  (307.46,449.43) | 2.18 | 317.43  (253.46,390.84) | 2.47 | -0.16  (-0.37,0.12) |
| Trinidad and Tobago | 11.28  (10.48,12.06) | 1.76 | 4.49  (3.41,5.78) | 1.82 | -0.60  (-0.70,-0.48) |  | 12.04  (11.16,12.87) | 1.74 | 4.54  (3.46,5.81) | 1.79 | -0.62  (-0.71,-0.51) |  | 251.59  (233.83,268.58) | 1.76 | 95.85  (72.12,125.94) | 1.84 | -0.62  (-0.72,-0.50) |
| Tunisia | 6.68  (5.56,7.91) | 1.63 | 5.57  (4.15,7.48) | 1.83 | -0.17  (-0.41,0.19) |  | 7.02  (5.86,8.29) | 1.66 | 5.19  (3.89,6.94) | 1.86 | -0.26  (-0.47,0.04) |  | 153.91  (127.68,183.56) | 1.52 | 111.48  (81.68,151.29) | 1.75 | -0.28  (-0.50,0.05) |
| Turkey | 19.88  (16.99,22.91) | 1.96 | 11.91  (9.51,14.60) | 2.07 | -0.40  (-0.53,-0.23) |  | 20.38  (17.45,23.47) | 1.95 | 10.75  (8.59,13.18) | 2.02 | -0.47  (-0.59,-0.32) |  | 525.21  (443.26,602.11) | 1.93 | 250.47  (198.71,308.54) | 2.14 | -0.52  (-0.63,-0.38) |
| Turkmenistan | 22.07  (21.10,23.03) | 2.24 | 9.35  (7.44,11.66) | 2.52 | -0.58  (-0.66,-0.47) |  | 22.42  (21.39,23.41) | 2.23 | 9.23  (7.35,11.45) | 2.56 | -0.59  (-0.67,-0.49) |  | 586.31  (560.72,612.55) | 2.27 | 238.99  (189.21,301.66) | 2.48 | -0.59  (-0.68,-0.49) |
| Tuvalu | 17.68  (14.28,21.50) | 1.81 | 13.25  (10.32,17.00) | 1.63 | -0.25  (-0.44,0.01) |  | 18.56  (15.08,22.49) | 1.77 | 13.67  (10.71,17.41) | 1.54 | -0.26  (-0.44,-0.02) |  | 472.16  (379.30,582.65) | 1.84 | 337.02  (254.30,443.62) | 1.77 | -0.29  (-0.48,-0.02) |
| Uganda | 9.74  (8.05,11.47) | 2.51 | 8.16  (6.59,9.84) | 2.26 | -0.16  (-0.34,0.07) |  | 10.47  (8.71,12.32) | 2.52 | 8.64  (7.02,10.41) | 2.24 | -0.17  (-0.34,0.04) |  | 248.98  (200.21,299.93) | 2.47 | 208.98  (165.28,259.05) | 2.28 | -0.16  (-0.36,0.11) |
| Ukraine | 30.37  (29.19,31.47) | 2.51 | 16.34  (13.70,19.23) | 2.75 | -0.46  (-0.55,-0.37) |  | 27.83  (26.80,28.82) | 2.50 | 13.46  (11.41,15.82) | 2.75 | -0.52  (-0.59,-0.43) |  | 769.93  (741.71,797.16) | 2.56 | 373.66  (314.19,444.85) | 2.71 | -0.51  (-0.60,-0.42) |
| United Arab Emirates | 14.29  (11.28,17.41) | 1.46 | 8.84  (6.91,10.98) | 1.80 | -0.38  (-0.55,-0.14) |  | 15.57  (12.24,18.75) | 1.53 | 9.23  (7.31,11.38) | 1.90 | -0.41  (-0.56,-0.19) |  | 307.53  (241.58,379.72) | 1.27 | 177.79  (134.49,225.85) | 1.57 | -0.42  (-0.59,-0.18) |
| United Kingdom | 13.46  (12.98,13.77) | 2.38 | 7.37  (6.16,8.79) | 2.19 | -0.45  (-0.54,-0.35) |  | 11.91  (11.42,12.19) | 2.18 | 5.62  (5.19,5.88) | 1.92 | -0.53  (-0.55,-0.51) |  | 240.69  (234.05,244.87) | 2.29 | 107.39  (102.01,111.52) | 1.94 | -0.55  (-0.57,-0.54) |
| United Republic of Tanzania | 9.76  (8.23,11.48) | 1.81 | 7.18  (5.84,8.56) | 1.60 | -0.26  (-0.42,-0.09) |  | 10.48  (8.84,12.31) | 1.85 | 7.68  (6.29,9.16) | 1.62 | -0.27  (-0.41,-0.09) |  | 251.28  (208.06,298.61) | 1.76 | 180.45  (143.00,221.12) | 1.57 | -0.28  (-0.44,-0.09) |
| United States of America | 8.31  (7.98,8.52) | 2.10 | 5.89  (5.10,6.87) | 1.93 | -0.29  (-0.39,-0.18) |  | 5.80  (5.51,5.95) | 2.04 | 3.40  (3.19,3.54) | 1.85 | -0.41  (-0.43,-0.39) |  | 125.98  (122.34,128.46) | 2.07 | 75.73  (72.84,78.30) | 1.84 | -0.40  (-0.42,-0.38) |
| United States Virgin Islands | 11.11  (9.50,12.96) | 2.16 | 10.32  (8.65,12.03) | 3.43 | -0.07  (-0.26,0.16) |  | 11.34  (9.71,13.18) | 2.11 | 9.79  (8.29,11.34) | 3.25 | -0.14  (-0.30,0.07) |  | 255.70  (217.51,299.17) | 2.28 | 218.79  (177.94,262.17) | 3.78 | -0.14  (-0.33,0.11) |
| Uruguay | 17.71  (16.86,18.59) | 2.36 | 11.34  (8.93,14.19) | 2.37 | -0.36  (-0.50,-0.20) |  | 17.85  (16.95,18.72) | 2.28 | 10.66  (9.78,11.49) | 2.29 | -0.40  (-0.45,-0.35) |  | 394.45  (377.89,412.79) | 2.39 | 234.64  (216.95,251.71) | 2.38 | -0.41  (-0.45,-0.36) |
| Uzbekistan | 22.32  (21.22,23.37) | 2.29 | 13.46  (11.39,15.56) | 1.92 | -0.40  (-0.49,-0.30) |  | 22.38  (21.27,23.44) | 2.28 | 13.43  (11.44,15.44) | 1.93 | -0.40  (-0.49,-0.31) |  | 605.88  (577.35,633.47) | 2.32 | 338.07  (283.81,393.68) | 1.88 | -0.44  (-0.53,-0.35) |
| Vanuatu | 15.85  (11.56,20.60) | 1.95 | 14.99  (11.05,19.54) | 1.86 | -0.05  (-0.29,0.28) |  | 16.88  (12.22,21.57) | 1.88 | 15.75  (11.67,20.34) | 1.78 | -0.07  (-0.28,0.24) |  | 415.33  (300.21,552.71) | 2.11 | 395.94  (289.07,520.86) | 2.05 | -0.05  (-0.31,0.32) |
| Venezuela  (Bolivarian Republic of) | 19.58  (18.39,20.55) | 1.63 | 13.94  (10.57,17.87) | 1.68 | -0.29  (-0.46,-0.08) |  | 20.09  (18.77,21.13) | 1.65 | 12.58  (9.63,16.01) | 1.78 | -0.37  (-0.52,-0.20) |  | 448.73  (426.09,469.84) | 1.71 | 286.06  (215.61,369.98) | 1.80 | -0.36  (-0.52,-0.17) |
| Viet Nam | 17.69  (14.14,21.31) | 2.38 | 10.59  (8.51,12.64) | 2.81 | -0.40  (-0.55,-0.19) |  | 18.40  (14.65,22.05) | 2.39 | 9.98  (8.14,11.84) | 2.78 | -0.46  (-0.58,-0.27) |  | 458.91  (362.14,559.74) | 2.30 | 240.08  (190.13,293.91) | 2.72 | -0.48  (-0.62,-0.29) |
| Zimbabwe | 12.44  (10.89,13.98) | 1.11 | 13.11  (10.24,16.39) | 1.02 | 0.05  (-0.19,0.35) |  | 13.10  (11.47,14.73) | 1.13 | 13.71  (10.79,17.15) | 1.05 | 0.05  (-0.19,0.31) |  | 300.08  (260.61,340.73) | 1.16 | 326.93  (251.59,415.13) | 1.07 | 0.09  (-0.18,0.39) |
| Zimbabwe | 12.44  (10.89,13.98) | 1.11 | 13.11  (10.24,16.39) | 1.02 | 0.05  (-0.19,0.35) |  | 13.11  (11.48,14.74) | 1.13 | 13.72  (10.80,17.16) | 1.05 | 0.05  (-0.19,0.32) |  | 300.08  (260.61,340.74) | 1.16 | 326.93  (251.59,415.14) | 1.07 | 0.09  (-0.18,0.40) |
| Zimbabwe | 12.44  (10.89,13.98) | 1.11 | 13.11  (10.24,16.39) | 1.02 | 0.05  (-0.19,0.35) |  | 13.12  (11.49,14.75) | 1.13 | 13.73  (10.81,17.17) | 1.05 | 0.05  (-0.19,0.33) |  | 300.08  (260.61,340.75) | 1.16 | 326.93  (251.59,415.15) | 1.07 | 0.09  (-0.18,0.41) |

ASIR, age standardized incident rate; ASMR, age standardized mortality rate; DALY, disability adjusted life-year; eAPC: estimated annual percentage change; CI: confidence interval.
