# Supplementary material for: Chinese and global burdens of gastric cancer from 1990 to 2019
Source: Cancer Med. 2021 May 1;10(10):3461–73. doi: 10.1002/cam4.3892 (PMC8124120; doi:10.1002/cam4.3892)
Supplement: Supplementary file 3 — Table S3 [file CAM4-10-3461-s002.docx]

**Table S3. The** **age-specific rates of gastric cancer in China in 2019.**

| **Characteristic** | **Age-specific incident rate (per 100,000 persons)** | | |  | **Age-specific mortality rate (per 100,000 persons)** | | |  | **Age-specific DALY rate (per 100,000 persons)** | | |
| --- | --- | --- | --- | --- | --- | --- | --- | --- | --- | --- | --- |
|  | Both (95%CI) | Male (95%CI) | Female (95%CI) |  | Both (95%CI) | Male (95%CI) | Female (95%CI) |  | Both (95%CI) | Male (95%CI) | Female (95%CI) |
| 0-14 y | 0.00  (0.00,0.00) | 0.00  (0.00,0.00) | 0.00  (0.00,0.00) |  | 0.00  (0.00,0.00) | 0.00  (0.00,0.00) | 0.00  (0.00,0.00) |  | 0.00  (0.00,0.00) | 0.00  (0.00,0.00) | 0.00  (0.00,0.00) |
| 15-19 y | 0.43  (0.36,0.51) | 0.54  (0.42,0.68) | 0.31  (0.26,0.38) |  | 0.13  (0.11,0.15) | 0.15  (0.12,0.19) | 0.10  (0.08,0.12) |  | 7.69  (6.91,8.59) | 7.43  (6.54,8.57) | 7.96  (6.92,9.12) |
| 20-24 y | 1.32  (1.10,1.56) | 1.60  (1.26,1.98) | 1.01  (0.77,1.28) |  | 0.43  (0.37,0.51) | 0.50  (0.40,0.61) | 0.36  (0.27,0.45) |  | 19.74  (17.92,21.78) | 18.37  (16.50,20.55) | 21.15  (18.53,24.06) |
| 25-29 y | 2.49  (2.08,2.93) | 2.94  (2.41,3.55) | 2.01  (1.49,2.59) |  | 0.90  (0.76,1.05) | 1.02  (0.84,1.22) | 0.78  (0.58,1.00) |  | 36.84  (33.72,40.12) | 35.50  (32.52,39.34) | 38.20  (33.76,43.12) |
| 30-34 y | 5.67  (4.79,6.63) | 7.47  (6.16,8.93) | 3.83  (2.89,4.92) |  | 2.17  (1.86,2.53) | 2.75  (2.28,3.32) | 1.57  (1.19,2.01) |  | 78.97  (72.75,85.60) | 85.32  (77.91,94.44) | 72.52  (64.19,81.04) |
| 35-39 y | 9.30  (7.82,10.91) | 12.79  (10.21,15.46) | 5.68  (4.36,7.17) |  | 4.18  (3.53,4.87) | 5.57  (4.48,6.72) | 2.73  (2.08,3.48) |  | 131.16  (120.88,142.45) | 145.12  (130.83,160.12) | 116.98  (104.83,129.78) |
| 40-44 y | 16.98  (13.93,20.36) | 24.65  (19.41,31.07) | 8.98  (6.96,11.30) |  | 7.80  (6.42,9.25) | 11.07  (8.72,13.83) | 4.40  (3.42,5.60) |  | 220.90  (202.23,239.82) | 262.12  (234.99,292.85) | 178.99  (159.46,199.64) |
| 45-49 y | 23.60  (18.88,29.16) | 35.37  (26.76,46.06) | 11.38  (8.77,14.55) |  | 12.13  (9.81,14.78) | 17.85  (13.58,22.97) | 6.19  (4.78,7.82) |  | 325.59  (294.42,357.30) | 424.53  (370.61,483.66) | 225.42  (203.10,251.16) |
| 50-54 y | 39.70  (31.68,48.61) | 61.12  (46.14,79.18) | 18.08  (14.05,22.91) |  | 22.08  (17.76,26.96) | 33.34  (25.15,42.88) | 10.73  (8.37,13.56) |  | 517.96  (463.42,570.95) | 703.16  (606.89,805.17) | 334.15  (296.06,379.43) |
| 55-59 y | 65.70  (52.44,81.04) | 102.32  (77.70,132.50) | 28.73  (22.37,36.42) |  | 38.21  (30.78,46.88) | 58.12  (43.86,74.61) | 18.12  (14.07,23.11) |  | 718.77  (646.49,791.86) | 1020.43  (884.79,1154.48) | 425.90  (382.26,478.54) |
| 60-64 y | 101.31  (82.65,123.15) | 156.75  (121.78,199.60) | 45.34  (35.81,56.56) |  | 62.62  (51.04,75.88) | 94.41  (72.73,119.73) | 30.53  (24.18,38.21) |  | 969.41  (878.31,1065.81) | 1391.14  (1224.61,1569.06) | 569.31  (512.62,635.84) |
| 65-69 y | 143.29  (118.33,171.98) | 220.22  (172.15,275.69) | 69.12  (55.88,85.20) |  | 94.59  (78.11,112.51) | 141.78  (110.60,176.28) | 49.10  (39.41,60.77) |  | 1226.47  (1109.04,1345.67) | 1762.59  (1539.69,1987.88) | 735.44  (660.41,819.15) |
| 70-74 y | 211.22  (177.00,250.24) | 322.70  (254.92,396.35) | 105.20  (85.54,129.99) |  | 150.45  (125.31,177.15) | 223.59  (176.88,275.59) | 80.90  (65.30,99.08) |  | 1465.77  (1334.99,1597.47) | 2111.10  (1862.75,2371.11) | 891.38  (797.36,991.72) |
| 75-79 y | 239.42  (203.68,279.20) | 361.22  (290.89,440.54) | 129.82  (105.35,159.23) |  | 196.41  (166.22,227.73) | 288.32  (233.89,347.14) | 113.70  (92.59,139.69) |  | 1529.10  (1399.23,1647.47) | 2173.53  (1951.28,2394.77) | 1001.32  (887.86,1110.19) |
| 80+ y | 268.03  (228.35,303.60) | 435.05  (368.97,504.25) | 161.65  (128.71,193.39) |  | 264.89  (229.02,297.86) | 411.30  (345.04,475.41) | 171.63  (136.96,203.68) |  | 1470.51  (1278.34,1589.33) | 2102.19  (1870.60,2276.96) | 1069.07  (874.32,1188.01) |

DALY, disability adjusted life-year; CI: confidence interval.
