# Supplementary material for: Chinese and global burdens of gastric cancer from 1990 to 2019
Source: Cancer Med. 2021 May 1;10(10):3461–73. doi: 10.1002/cam4.3892 (PMC8124120; doi:10.1002/cam4.3892)
Supplement: Supplementary file 4 — Table S4 [file CAM4-10-3461-s003.docx]

**Table S4. The age-specific rates of gastric cancer globally in 2019.**

| **Characteristic** | **Age-specific incident rate (per 100,000 persons)** | | |  | **Age-specific mortality rate (per 100,000 persons)** | | |  | **Age-specific DALY rate (per 100,000 persons)** | | |
| --- | --- | --- | --- | --- | --- | --- | --- | --- | --- | --- | --- |
|  | Both (95%CI) | Male (95%CI) | Female (95%CI) |  | Both (95%CI) | Male (95%CI) | Female (95%CI) |  | Both (95%CI) | Male (95%CI) | Female (95%CI) |
| 0-14 y | 0.00  (0.00,0.00) | 0.00  (0.00,0.00) | 0.00  (0.00,0.00) |  | 0.00  (0.00,0.00) | 0.00  (0.00,0.00) | 0.00  (0.00,0.00) |  | 0.00  (0.00,0.00) | 0.00  (0.00,0.00) | 0.00  (0.00,0.00) |
| 15-19 y | 0.21  (0.19,0.23) | 0.22  (0.19,0.25) | 0.21  (0.18,0.23) |  | 0.11  (0.10,0.12) | 0.10  (0.09,0.12) | 0.11  (0.10,0.13) |  | 7.69  (6.91,8.59) | 7.43  (6.54,8.57) | 7.96  (6.92,9.12) |
| 20-24 y | 0.56  (0.51,0.61) | 0.56  (0.49,0.63) | 0.56  (0.49,0.63) |  | 0.29  (0.27,0.32) | 0.27  (0.25,0.31) | 0.32  (0.28,0.36) |  | 19.74  (17.92,21.78) | 18.37  (16.50,20.55) | 21.15  (18.53,24.06) |
| 25-29 y | 1.11  (1.01,1.21) | 1.14  (1.03,1.29) | 1.08  (0.94,1.23) |  | 0.59  (0.54,0.64) | 0.57  (0.52,0.63) | 0.61  (0.54,0.69) |  | 36.84  (33.72,40.12) | 35.50  (32.52,39.34) | 38.20  (33.76,43.12) |
| 30-34 y | 2.54  (2.33,2.78) | 2.92  (2.60,3.28) | 2.16  (1.89,2.45) |  | 1.38  (1.27,1.50) | 1.49  (1.36,1.65) | 1.27  (1.12,1.42) |  | 78.97  (72.75,85.60) | 85.32  (77.91,94.44) | 72.52  (64.19,81.04) |
| 35-39 y | 4.12  (3.78,4.51) | 4.82  (4.27,5.40) | 3.41  (3.06,3.79) |  | 2.51  (2.32,2.73) | 2.78  (2.51,3.07) | 2.24  (2.01,2.49) |  | 131.16  (120.88,142.45) | 145.12  (130.83,160.12) | 116.98  (104.83,129.78) |
| 40-44 y | 7.59  (6.85,8.39) | 9.51  (8.28,10.91) | 5.64  (5.05,6.25) |  | 4.67  (4.27,5.08) | 5.54  (4.95,6.18) | 3.79  (3.38,4.23) |  | 220.90  (202.23,239.82) | 262.12  (234.99,292.85) | 178.99  (159.46,199.64) |
| 45-49 y | 12.21  (10.87,13.72) | 16.47  (14.07,19.36) | 7.89  (7.02,8.81) |  | 7.68  (6.94,8.44) | 10.01  (8.72,11.40) | 5.33  (4.80,5.95) |  | 325.59  (294.42,357.30) | 424.53  (370.61,483.66) | 225.42  (203.10,251.16) |
| 50-54 y | 21.20  (18.75,23.89) | 29.80  (25.23,35.20) | 12.67  (11.20,14.21) |  | 13.77  (12.35,15.21) | 18.69  (16.13,21.42) | 8.90  (7.89,10.12) |  | 517.96  (463.42,570.95) | 703.16  (606.89,805.17) | 334.15  (296.06,379.43) |
| 55-59 y | 32.77  (29.22,36.93) | 47.91  (41.12,55.89) | 18.06  (16.18,20.26) |  | 21.85  (19.62,24.08) | 31.00  (26.82,35.13) | 12.97  (11.65,14.62) |  | 718.77  (646.49,791.86) | 1020.43  (884.79,1154.48) | 425.90  (382.26,478.54) |
| 60-64 y | 49.47  (44.39,55.51) | 73.17  (63.64,84.88) | 26.98  (24.26,30.09) |  | 34.30  (30.97,37.71) | 49.18  (43.25,55.53) | 20.19  (18.19,22.57) |  | 969.41  (878.31,1065.81) | 1391.14  (1224.61,1569.06) | 569.31  (512.62,635.84) |
| 65-69 y | 72.04  (64.99,80.46) | 106.53  (92.29,123.35) | 40.44  (36.38,45.36) |  | 51.69  (46.76,56.70) | 74.19  (64.61,83.88) | 31.07  (27.90,34.69) |  | 1226.47  (1109.04,1345.67) | 1762.59  (1539.69,1987.88) | 735.44  (660.41,819.15) |
| 70-74 y | 100.43  (90.64,111.13) | 149.00  (130.22,170.42) | 57.19  (51.00,64.03) |  | 75.41  (68.58,82.27) | 108.48  (95.33,121.97) | 45.98  (41.17,51.18) |  | 1465.77  (1334.99,1597.47) | 2111.10  (1862.75,2371.11) | 891.38  (797.36,991.72) |
| 75-79 y | 119.80  (108.47,130.71) | 175.35  (155.86,196.45) | 74.30  (65.91,82.30) |  | 99.41  (90.96,107.22) | 141.00  (126.18,155.35) | 65.35  (57.79,72.57) |  | 1529.10  (1399.23,1647.47) | 2173.53  (1951.28,2394.77) | 1001.32  (887.86,1110.19) |
| 80+ y | 154.03  (130.33,169.05) | 222.10  (193.49,243.35) | 110.77  (89.33,125.23) | | 147.35  (126.62,159.80) | 204.46  (181.00,221.44) | 111.06  (89.98,123.67) | | 1470.51  (1278.34,1589.33) | 2102.19  (1870.60,2276.96) | 1069.07  (874.32,1188.01) |

DALY, disability adjusted life-year; CI: confidence interval.
