# Supplementary material for: Chinese and global burdens of gastric cancer from 1990 to 2019
Source: Cancer Med. 2021 May 1;10(10):3461–73. doi: 10.1002/cam4.3892 (PMC8124120; doi:10.1002/cam4.3892)
Supplement: Supplementary file 5 — Table S5 [file CAM4-10-3461-s001.docx]

**Table S5: eAPC in age-specific incident rates by genders in China from 1990 to 2019**

| **Characteristics** | **Both genders** | **Males** | **Females** |
| --- | --- | --- | --- |
|  | eAPC (95%CI) | eAPC (95%CI) | eAPC (95%CI) |
| 0-4 years | 0.00(0.00,0.00) | 0.00(0.00,0.00) | 0.00(0.00,0.00) |
| 5-14 years | 0.00(0.00,0.00) | 0.00(0.00,0.00) | 0.00(0.00,0.00) |
| 15-49 years | 0.30(0.02,0.63) | 0.54(0.14,1.10) | -0.08(-0.35,0.27) |
| 50-69 years | -0.29(-0.44,-0.09) | -0.19(-0.40,0.11) | -0.48(-0.61,-0.31) |
| 70+ years | -0.08(-0.24,0.10) | 0.02(-0.20,0.32) | -0.29(-0.45,-0.10) |

eAPC: estimated Annual percent change; CI, confidence interval
